# Molecular changes associated with spontaneous phenotypic variation of *Paenibacillus polymyxa*, a commonly used biocontrol agent, and temperature-dependent control of variation

**DOI:** 10.1038/s41598-020-73716-7

**Published:** 2020-10-06

**Authors:** Younmi Lee, Young Soo Kim, Kotnala Balaraju, Young-Su Seo, Jungwook Park, Choong-Min Ryu, Seung-Hwan Park, Jihyun F. Kim, Seogchan Kang, Yongho Jeon

**Affiliations:** 1 Department of Plant Medicals, Andong National University, Andong, 36729 Republic of Korea; 2 Agricultural Science and Technology Research Institute, Andong National University, Andong, 36729 Republic of Korea; 3 Department of Microbiology, Pusan National University, Pusan, 46241 Republic of Korea; 4 Infectious Disease Research Centre, KRIBB, Daejeon, 34141 Republic of Korea; 5 Department of Biosystems and Bioengineering, KRIBB School of Biotechnology, Korea University of Science and Technology, Daejeon, 34141 Republic of Korea; 6 Department of Systems Biology, Division of Life Sciences, and Institute for Life Science and Biotechnology, Yonsei University, Seoul, 03722 Republic of Korea; 7 Strategic Initiative for Microbiomes in Agriculture and Food (iMAF), Yonsei University, Seoul, 03722 Republic of Korea; 8 Department of Plant Pathology and Environmental Microbiology, Pennsylvania State University, University Park, PA 16802 USA

**Keywords:** Biotechnology, Microbiology

## Abstract

There has been a growing interest in deploying plant growth-promoting rhizobacteria (PGPR) as a biological control agent (BCA) to reduce the use of agrochemicals. Spontaneous phenotypic variation of PGPR, which causes the loss of traits crucial for biocontrol, presents a large obstacle in producing commercial biocontrol products. Here, we report molecular changes associated with phenotypic variation in *Paenibacillus polymyxa*, a PGPR widely used for biocontrol worldwide, and a simple cultural change that can prevent the variation. Compared to B-type (non-variant) cells of *P. polymyxa* strain E681, its phenotypic variant, termed as F-type, fails to form spores, does not confer plant growth-promoting effect, and displays altered colony and cell morphology, motility, antagonism against other microbes, and biofilm formation. This variation was observed in all tested strains of *P. polymyxa*, but the frequency varied among them. RNA-seq analysis revealed differential regulation of many genes involved in sporulation, flagella synthesis, carbohydrate metabolism, and antimicrobial production in F-type cells, consistent with their pleiotropic phenotypic changes. F-type cells's sporulation was arrested at stage 0, and the key sporulation gene *spo0A* was upregulated only in B-type cells. The phenotypic variation could be prevented by altering the temperature for growth. When E681 was cultured at 20 °C or lower, it exhibited no variation for 7 days and still reached ~ 10^8^ cfu/mL, the level sufficient for commercial-scale production of biocontrol products.

## Introduction

The heavy reliance on chemical pesticides and fertilizers to maximize crop production has adverse effects on human health and the environment^1–3^. Eco-friendly farming strategies have been promoted to mitigate this growing global problem. One strategy is applying specific microorganisms that enhance plant growth and health. Plant growth-promoting rhizobacteria (PGPR) potentially serve as an environmentally sustainable alternative to agrochemicals^4–7^ thanks to their well-known ability to colonize plant roots, stimulate plant growth, and reduce disease incidence in crop plants^8–12^. *Paenibacillus* spp., well-known PGPR, promote plant growth and protect plants from various phytopathogenic bacteria, fungi, nematodes, and viruses via multiple mechanisms^13–15^. One mechanism is producing numerous secondary metabolites that suppress diverse phytopathogens^16^. Besides, the long shelf-life of *Paenibacillus* spp. makes them suitable for formulating commercial biocontrol products^17^. Especially, *P. polymyxa* has been frequently used as a biological control agent (BCA) globally due to its rhizosphere competence and tolerance to harsh environmental conditions^6,18^.

Some *Paenibacillus* spp. have been shown to display phenotypic variation, including reversible switching between the rod- and coccoid-shaped cells^19,20^. A recent study showed that *P. polymyxa* also formed two forms of distinctly shaped colonies when cultured on a solid medium, presumably without involving any genetic alterations^15^. Such microbial phenotypic variation appears to be a survival strategy under varying environmental conditions^20,21^. When environmental conditions change, microorganisms trigger a set of complex regulatory networks that drive cellular or developmental changes needed for their adaptation to the environment or long-term survival.

Here, we investigated the molecular basis of phenotypic variation using *P. polymyxa* strain E681. This strain was isolated from the roots of winter barley in Korea and has been shown to colonize the rhizospheres of cucumbers, pepper, sesame, and *Arabidopsis thaliana* and to benefit plants in vitro and in vivo^22,23^. Gene expression patterns in B-type (non-variant) and F-type (variant) cells were compared via RNA-seq to identify likely gene expression changes underpinning observed phenotypic changes. We also attempted to develop simple strategies to prevent or minimize phenotypic variation during mass production. A large proportion of E681 cells converted from B-type to F-type before lysis in liquid culture. The phenotypic variation of E681 affected spore production, a trait required for commercialization, and the production of antimicrobials. The phenotypic variation also occurred when E681 cells were applied in the field, likely reducing its efficacy as a biocontrol agent (BCA). This variation was not unique to E681, with other *P. polymyxa* strains also displaying varying variation frequencies. Bacterial growth conditions (e.g., pH, temperature, the type and amount of specific nutrients) likely affect the phenotypic variation. We report that growth temperature is a crucial regulator for phenotypic variation in *P. polymyxa*.

## Results

### Phenotypic variation of *Paenibacillus polymyxa *occurs in culture and *in planta*

**Figure 1:**
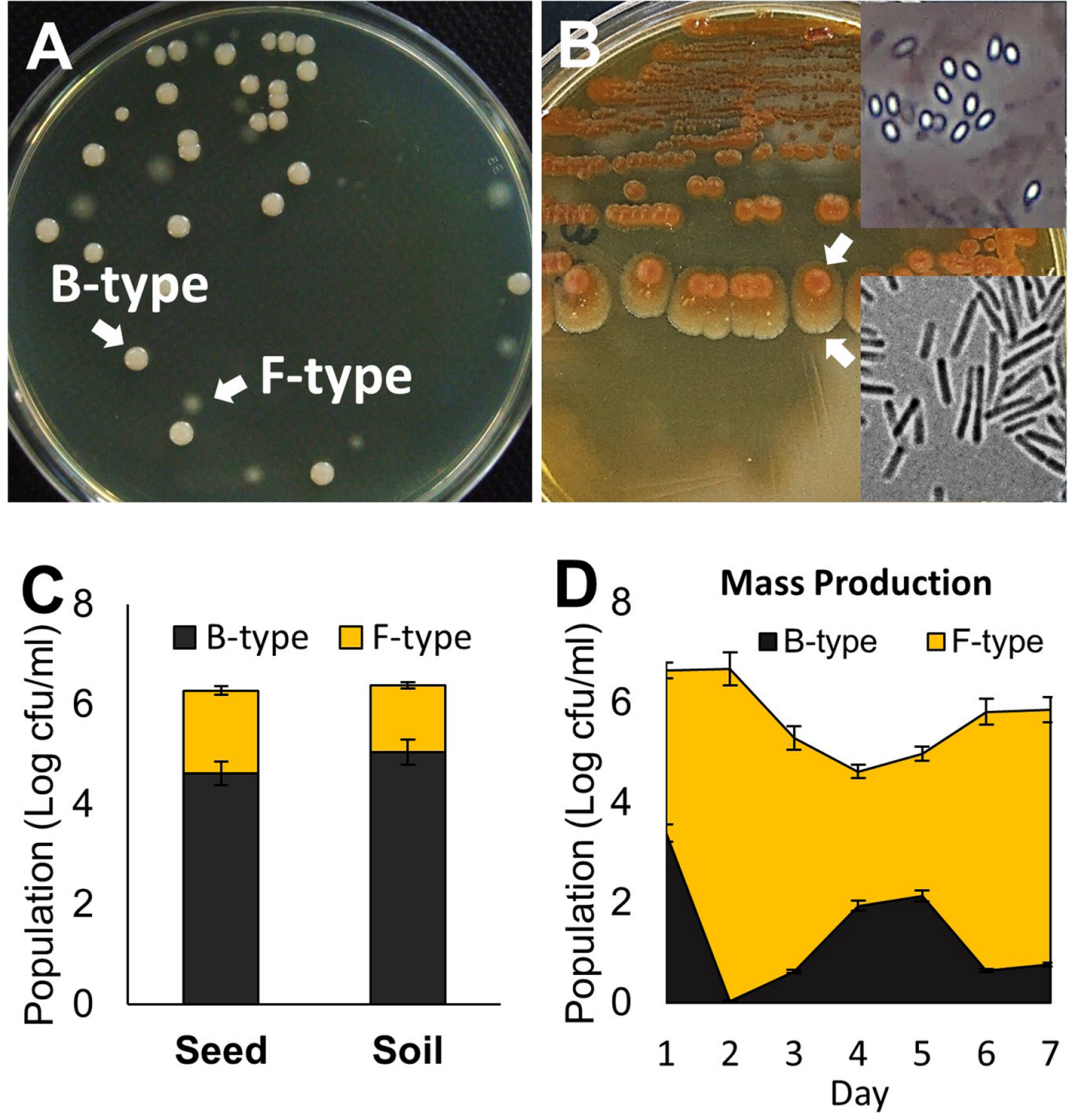
Phenotypic variation of *P. polymyxa* in culture. (**A**) Cells in 4-day-old TSB culture formed two distinct types of colonies, termed as B (wild-type) and F (variant) types when plated on TSA and incubated at 28 °C for 2 days. (**B**) Phenotypic variation was also observed when cells in B-type colonies were streaked on TSA plates and cultured at 37 °C for 10 days. The production of endospores was observed at the center of the colony using a phase-contrast microscope. The outermost region of this colony lacks endospores. (**C**) The phenotypic variation observed on cucumber seedlings when they were treated with B-type cell suspensions (10^6^ cfu/mL) via seed coating or soil treatment methods and incubated for 11 days at 28 °C. (**D**) The proportion of B- and F- type cells were determined during a large scale culture under the conditions employed for commercial biocontrol production preparation. After inoculating 1.5 L of 48-h-old culture initiated using B-type cells into 250 L fermenter containing a medium used for preparing industrial biocontrol products, samples taken every day for 7 days were analyzed.

When cells from the 3-day-old culture of *P. polymyxa* strain E681 were cultured in tryptic soy broth (TSB) at 30 °C were plated on tryptic soy agar (TSA), two distinct types of colonies were observed (Fig. 1A). While some colonies were bald, opaque, and milky-white with the shiny surface (B-type), others appeared flat, translucent, and round with a scalloped edge (F-type). This variation also occurred on a solid medium. When B-type cells were streaked on TSA and cultured at 37 °C, all colonies initially looked identical but later diverged into two types. At 10 days, some colonies had two distinct regions (Fig. 1B). The center region looked similar to B-type colonies, while the outer region was flat and translucent, similar to F-type colonies. A phenotypic variation occurred when cultured both in liquid and solid media. Phase-contrast imaging showed many endospores in the center region, but the outer region was composed of only vegetative cells. When cells in the outermost region were streaked on TSA, both types of colonies formed again.

This phenotypic variation occurred when cucumber seedlings were treated with B-type cells through seed coating or soil mixing methods (Fig. 1C). The degree of phenotypic variation from B-type to F-type during large-scale culturing was substantial (Fig. 1D). After inoculating 1.5 L of a 48-h-old culture of B-type cells into 250 L volume capacity fermenter containing a medium used for industrial-scale production of biocontrol products, we analyzed the composition of cells during culture at 28 °C for 7 days. The variation already occurred during the inoculum preparation stage. Although the population size of F-type cells fluctuated during mass culturing, they dominated.

### Characterization of phenotypic variation

**Figure 2:**
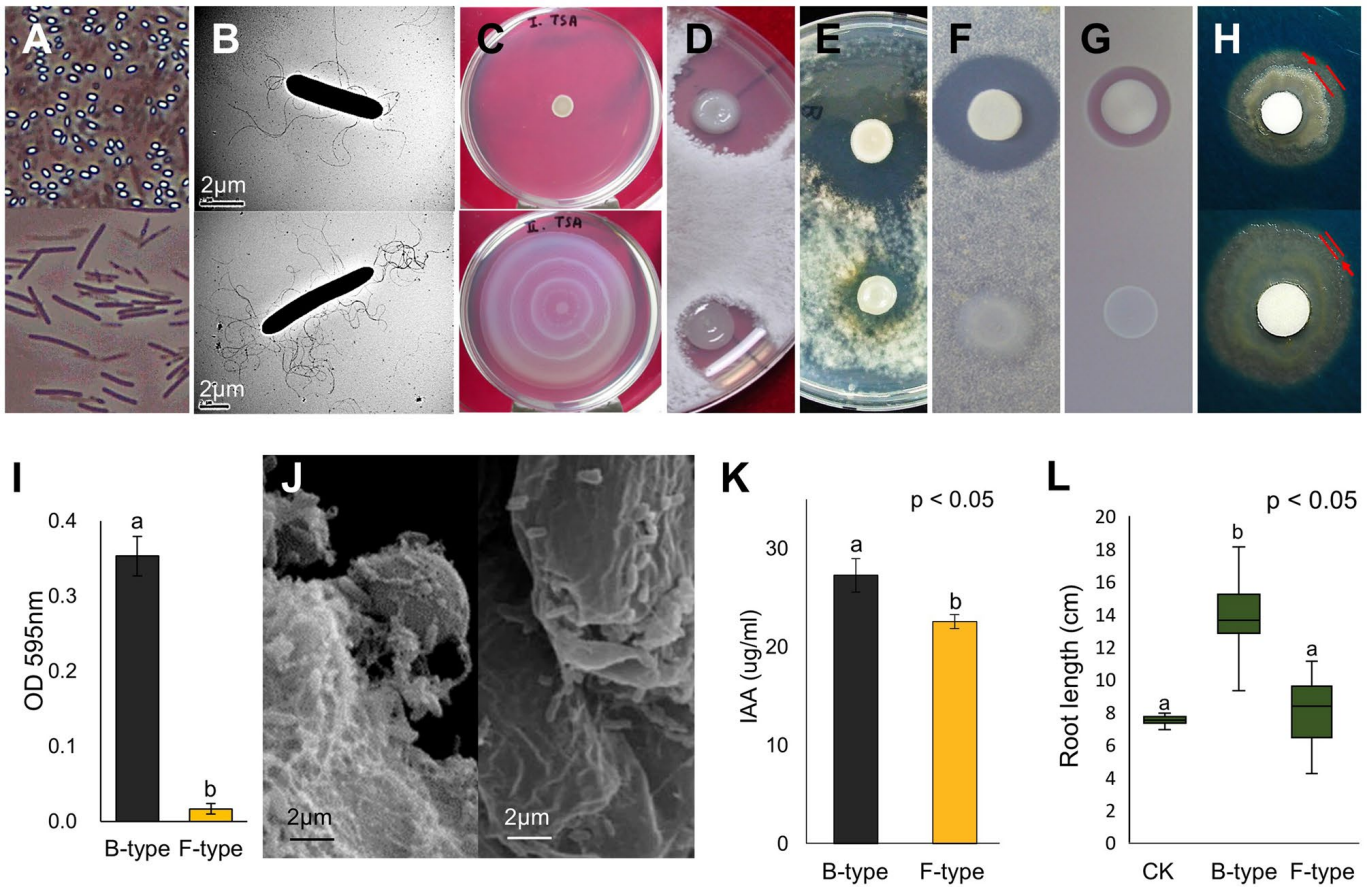
Characteristics of phenotypic variation in *P. polymyxa* E681 and the B- and F-type cells. Different characteristics between B- and F-types (**A**–**H**). Top row, B-type; bottom row, F-type. (**A**) Endospore formation and a large proportion of B-type cells had endospores, but no endospores were detected in F-type cells even after 9 days of culturing on TSA at 30 °C. (**B**) TEM imaging. The length of the F-type is longer than B-type and has more flagella. Scale bar = 2 µm. (**C**) Swarming motilities. B-type had no motility, but F-type showed a strong swarming response during 48 h of incubation at 30 °C on TSA medium containing 1.0% agar. (**D-F**) In vitro antagonism. B-type exhibited stronger antifungal activities against (**D**) *Rhizoctonia solani*, (**E**) *Cylindrocarpon destructans*, and (**F**) Antibacterial activity against *Escherichia coli* DH5α. (**G**) Protease activity. F-type showed no activity. (**H**) The iron chelating activity of siderophore. (**I**) Biofilm formation assay by crystal violet staining. A significant decrease in biofilm formation was observed in F-type compared to B-type. Bars with the same letters are not significantly different from each other, according to the least significant difference test (*p* < 0.05). (**J**) SEM imaging. Left, B-type; right, F-type. The B-type-treated cucumber root tip is covered with biofilm, while biofilm is hardly observed in the F-type-treated root tip. Effects of B- and F-type cells on (**K**) IAA production and (**L**) the growth of cucumber plants.

Besides their easily distinguishable colony morphologies, a significant difference in their ability to form endospores was observed. Many endospores were noted following a 2-day incubation of B-type cells on medium; however, no endospores were found when F-type cells were cultured under the same conditions (Fig. 2A). TEM demonstrated morphological differences in F-type cells (1.2 × 9.07 µm), which was thinner and longer than B-type cells (1.3 × 5.6 µm; Fig. 2B). The overall length-to-width (L/W) ratios were 4.66 µm and 7.20 µm for types B and F, respectively. Type ‘F’ bacteria had more flagella than type B, which correlated with increased swarming motility (Fig. 2C). In terms of antagonistic activity, B-type showed greater activity against *Rhizoctonia solani*, *Cylindrocarpon destructans,* and *Escherichia coli* than F-type (Fig. 2D–F). Protease activity was observed in B-type cell culture but was absent in F-type cell culture (Fig. 2G). The iron-chelating activity of siderophores in B-type showed a greater clear zone than that of F-type (Fig. 2H). B-type formed more biofilm than F-type during a 72-h long culturing (Fig. 2I). SEM imaging of inoculated roots showed that B-type treated cucumber root tips were covered with biofilm, while minimal biofilm was observed on F-type treated roots (Fig. 2J). Plant growth-promoting ability was also changed by phenotypic variation. In our experimental conditions, B-type showed a significantly increased level of IAA production (*p* < 0.05; 27.29 ± 1.69 µg/mL) compared to F-type (22.58 ± 0.69 µg/mL) (Fig. 2K). As for their growth-promoting effect, B-type treated cucumber seeds exhibited longer root length in comparison with F-treated seeds under in vitro conditions (Fig. 2L). The growth-promoting effect of F-type was indistinguishable from that of the control.

### Phenotypic variation is affected by growth temperature and strain selection

**Figure 3:**
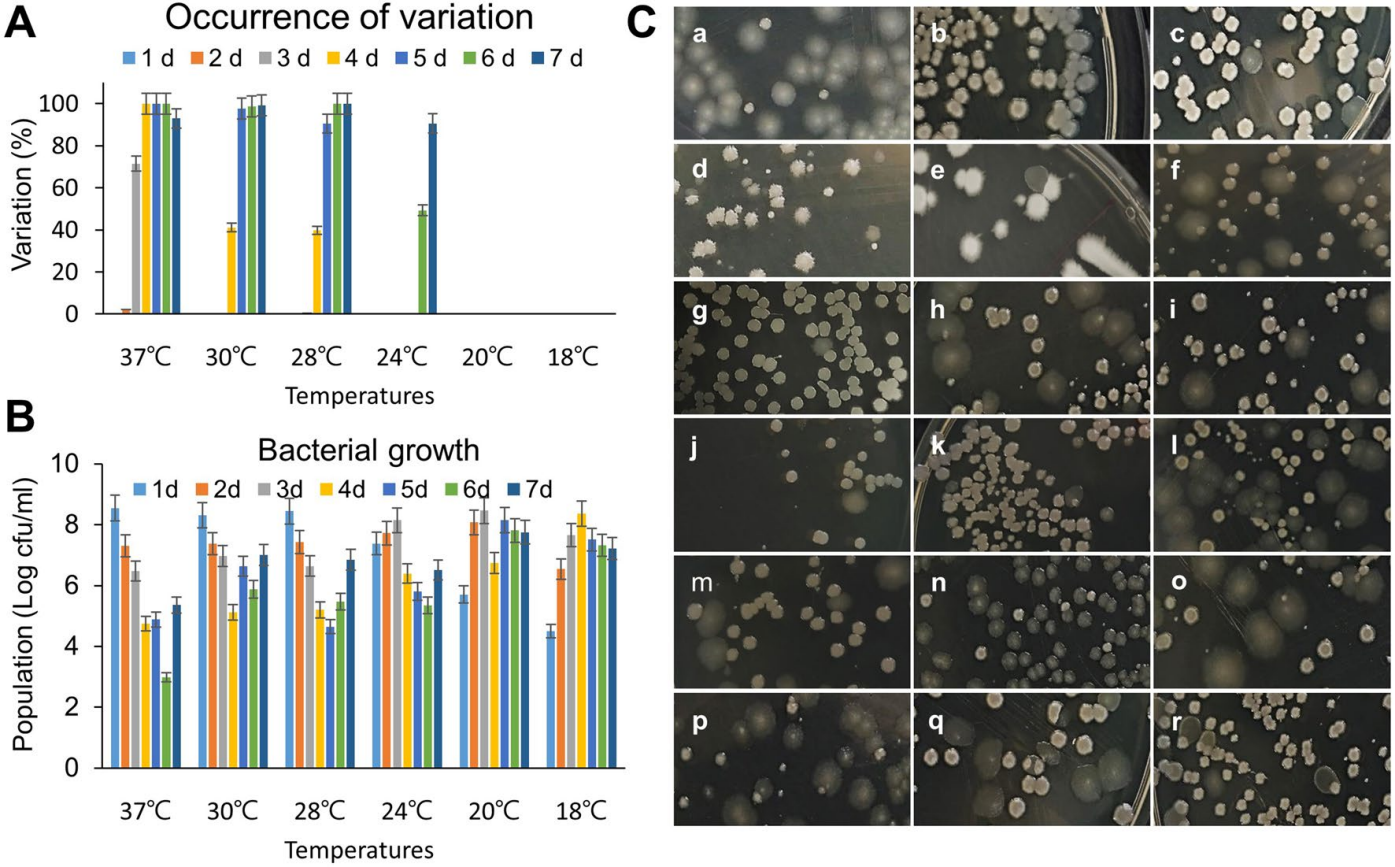
The occurrence of phenotypic variation among diverse *P. polymyxa* strains. (**A**) The phenotypic variation of B-type cells observed at various temperatures and incubation times. (**B**) The bacterial growth and population of B-type cells at different temperatures and durations of times for mass production. (**C**) Mixed colony morphologies in all the 18 strains of *P. polymyxa* (refer to Table 1).

The phenotypic variation of E681 was dependent on culture temperature (Fig. 3A). F-type colonies formed when incubated at 37, 30, 28, and 24 °C from the 2nd day to 4th day. However, no F-type colonies were observed when cultured at 20 °C and 18 °C during the 7-day culture period. There was also little difference in the bacterial population size with temperature (Fig. 3B). The bacterial populations were 8.1 Log cfu/mL and 8.5 Log cfu/mL at 24 °C and 20 °C, respectively on day 3, while the population was 8.4 Log cfu/mL at 18 °C on day 4. This result explains that even the lower temperature (18 °C) causes to increase in bacterial population (8 Log cfu/mL) as it appeared at a higher temperature (24 °C) in 3 days of incubation. A similar phenotypic variation was observed in all 18 *P. polymyxa* strains tested (Fig. 3C; Table 1). The 4-day old cultures displayed both types of colonies. However, the degree of phenotypic variation significantly varied among the strains. Strains E681 (B-type), GBR-1, and GBR-192 were converted into variants at a rate exceeding 90%, while the rate for C-3 and YGB-13 was nearly zero.

**Table 1:** Percentage of variation in all the strains.

Image (Fig. 3C)	Strain name	% Variation	References
a	E681 (B-type)	93.8	This study
b	C-1	0.6	Lab. strain
c	C-2	4.8	Lab. strain
d	C-3	0.0	Lab. strain
e	YGB-3	11.8	Lab. strain
f	YGB-4	24.9	Lab. strain
g	YGB-13	0.0	Lab. strain
h	YGB-14	29.2	Lab. strain
i	YGB-15	54.6	Lab. strain
j	YGB-17	0.8	Lab. strain
k	YGB-30	5.5	Lab. strain
l	GBR-540	60.1	Jeon et al.^55^
m	M125	12.3	Lab. strain
n	GBR-1	94.1	Kim et al.^56^
o	GBR-27	43.3	Son et al.^57^
p	GBR-192	90.0	Son et al.^57^
q	GBR-447	19.2	Son et al.^57^
r	GBR-462	0.9	Son et al.^57^

### Comparative transcriptomics

To investigate the molecular basis of phenotypic variation, genome-wide gene expression patterns in B- and F-type cells were analyzed via RNA-seq and compared. In total, 149 million paired-end reads were generated, of which 80 million were from B-type and 69 million from F-type cells (Table S1). The total reads of both types were mapped to the E681 reference genome (NC_014483.2), resulting in the alignment of 32 million reads from B-type and 56 million reads from F-type. The differentially expressed genes (DEGs) between B-type and F-type are plotted in Figure S1. Overall, there were 1,062 DEGs, 457 (9.5% of the total) upregulated, and 605 (12.6% of total) downregulated in F-type relative to B-type (Fig. S2). Sixteen randomly selected genes were analyzed via qPCR to confirm the RNA-Seq results. For comparison with the RNA-Seq data, qPCR relative expression was calculated as log2 fold changes in F-type relative to B-type. The results from RNA-seq and qPCR were compared using a scatter plot. The correlation coefficient (R^2^) was 0.798, supporting the realibility of the RNA-seq data (Fig. S3). DEGs were classified by gene ontology (GO) enrichment analysis to identify which functional groups are differentially expressed in the two types of cells (Table S2). DEGs were also annotated using KEGG pathway analysis, showing that DEGs with ≥ twofold change were associated using a *p*-value threshold of < 0.05 with 22 KEGG pathways (Table S3).

### Sporulation

**Figure 4:**
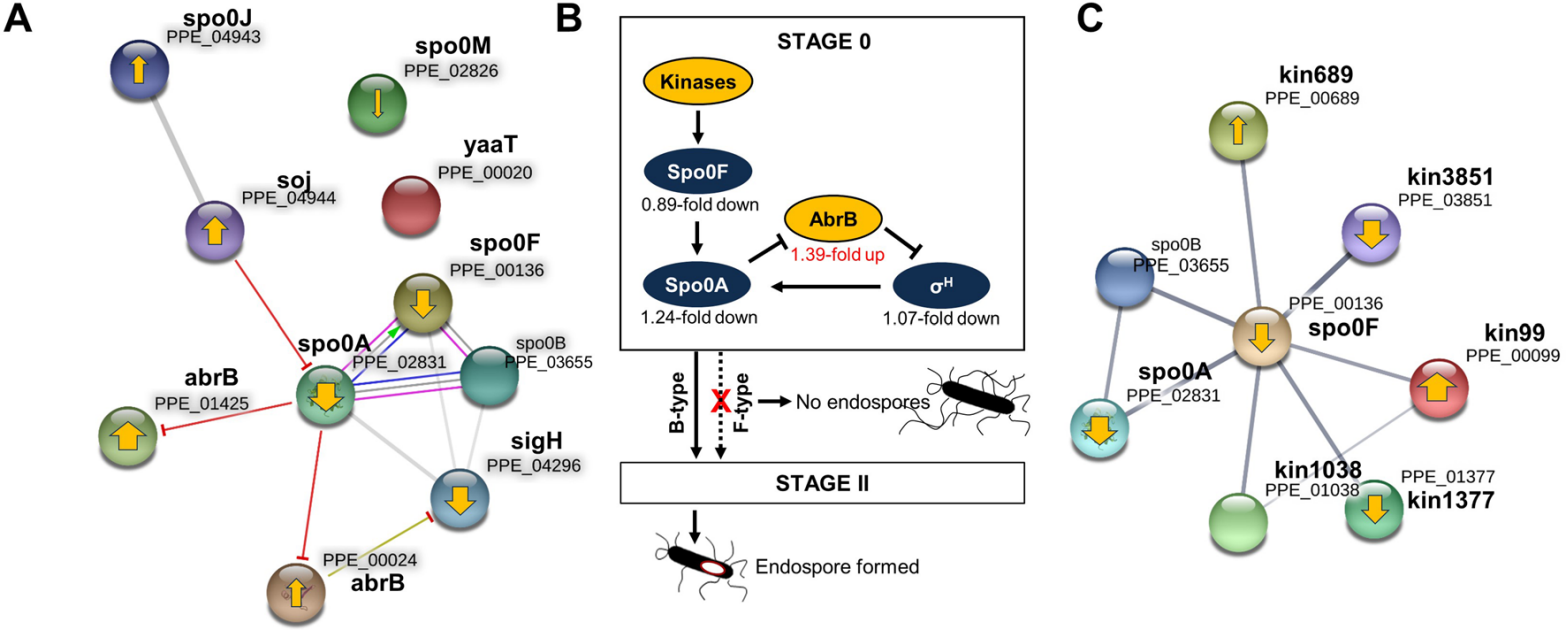
Downregulation of sporulation-related genes in F-type cells and a proposed model for sporulation is regulated. (**A**) The interactions between the genes likely to be expressed in sporulation stage 0 and regulatory genes. The STRING map of proteins expressed in sporulation stage 0. Line shape indicates the predicted mode of action. The thickness of the yellow arrows in each node represents the log_2_ fold changes of each gene by RNA-seq. The upward-pointing arrow indicates upregulation and downward direction indicates downregulation in F-type. A circle without a yellow arrow indicates no difference in the amount of expression between B- and F-types. Blunt ended arrows represent negative interaction. (**B**) A simplified schematic representation of the regulatory network that governs the initiation of sporulation in E681. Yellow represents key regulators of Spo0A. (**C**) The interaction network between kinases and Spo0F in E681 based on string analysis is shown. Line thickness indicates the strength of data support.

The terms related to sporulation identified through the GO analysis corresponded to ‘sporulation resulting in the formation of a cellular spore’ (GO:0030435), ‘asexual sporulation’ (GO:0030436), and ‘endospore-forming forespore’ (GO:0042601). Surprisingly, all the genes belonging to the three terms, except the *sigD* gene that encodes RNA polymerase sigma factor D, were downregulated in F-type (Table S4). This pattern may explain why endospores were not observed in F-type cells. The expression profiles of the 66 sporulation-specific genes were analysed, and the fold changes of many were negative (Fig. S4). Interestingly, the expression of stage 0 genes was downregulated less in F-type (fold change − 1) than B-type compared to the genes expressed at stages II–IV (fold change − 2 to − 8). The interactions between the genes expressed during sporulation stage 0 and regulatory genes were then analysed using *B*. *subtilis* as a model (Fig. 4A; Table S5). Accordingly, eight proteins (80%) were enriched in the same group, which was connected through functional interactions, and all of them were found to interact with Spo0A, Spo0F, Spo0B, and *SigH* positively. Regulator proteins, Soj (sporulation initiation inhibitor) and AbrB, interacted negatively with Spo0A and this corresponded with the opposite expression pattern. Based on the RNA-seq results, sporulation control in F-type was schematized (Fig. 4B). Two factors affecting the expression of *spo0A* were *abrB* and the kinases. The expression of the regulatory gene *abrB* was increased (log_2_ fold change, 1.39) in F-type, while *spo0A* (− 1.24) and *sigH* (− 1.07) were decreased. Kinase expression was another potential factor affecting the inhibition of *spo0A*. The STRING database was used to analyse protein–protein interactions between kinases and Spo0F (Fig. 4C; Table S6). In E681, five putative kinases, Kin99, Kin689, Kin1038, Kin1377, and Kin3851 have been reported^24^. The STRING map showed that all five kinases related to *Spo0F*. However, their expression patterns were complex. Of the five putative kinases, only *kin3851* and *kin1377* were downregulated in F-type.

### Flagella and chemotaxis

**Figure 5:**
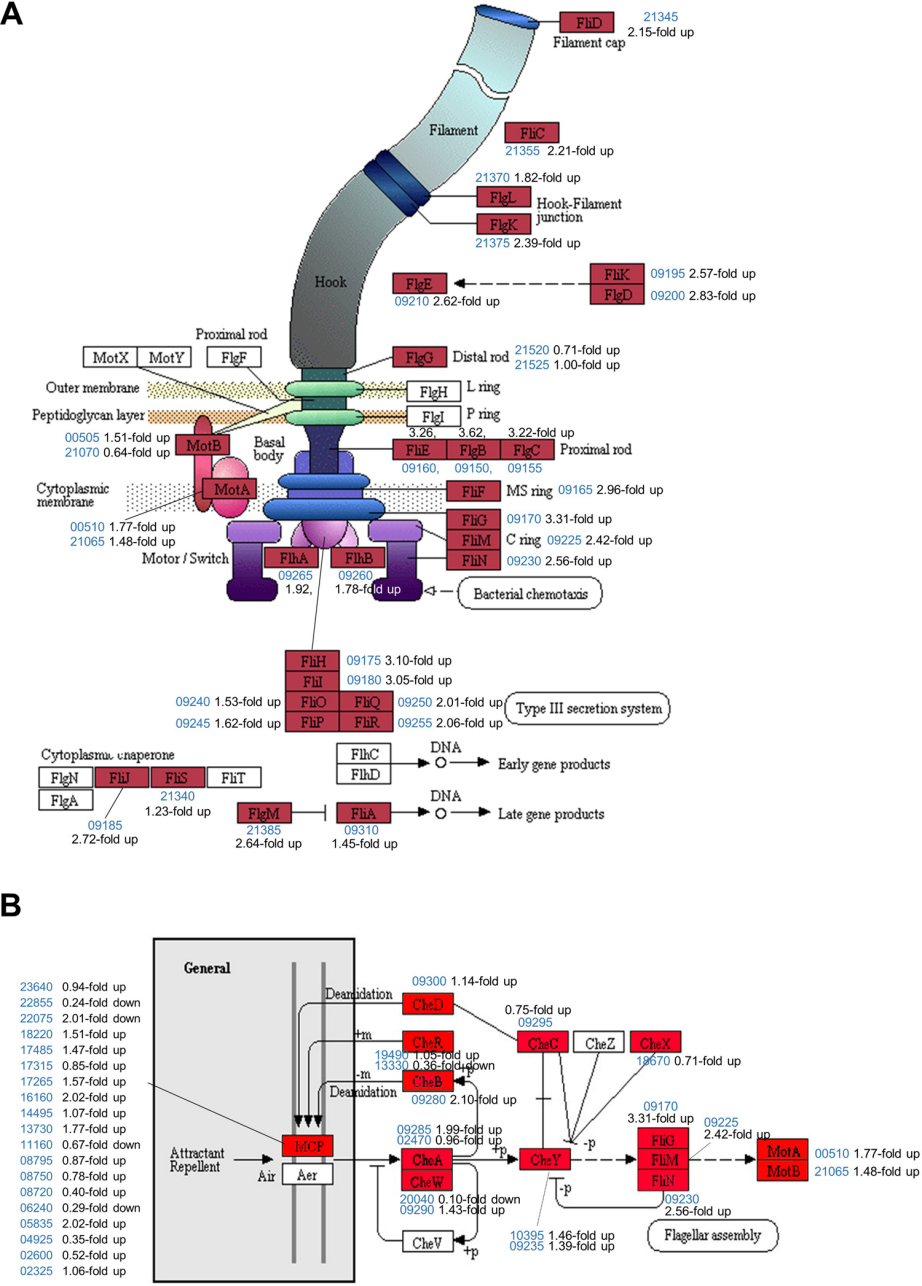
Upregulation of E681 genes likely involved in motility. Most genes related to flagella assembly (**A**) and bacterial chemotaxis (**B**) were overexpressed in F-type. The E681 genes are marked red boxes. The genes in uncolored boxes were not found in E681. The blue numbers are the gene IDs, with ‘PPE_RS’ omitted in front. The diagrams were adopted from the original KEGG pathway ppy02040 (**A**) and ppy02030 (**B**).

The most upregulated KEGG pathway was that of flagellar assembly (ppy02040). Interestingly, all 29 genes involved in the flagellar assembly were classified as DEGs and upregulated in F-type cells, and are highly flagellated (Fig. 2B, 5A; Table S7). Most genes involved in bacterial motility, such as the flagellar assembly, motor/switch, C-ring, rod, hook, and filament, were upregulated. Chemotaxis (ppy02030)-related genes were also upregulated in the KEGG pathway. Of the 25 genes in the pathway, 24 were upregulated (Fig. 5B; Table S7). These findings are consistent with an increase of flagella density and swarming motility of F-type cells relative to B-type cells.

### Carbohydrate metabolism

**Figure 6:**
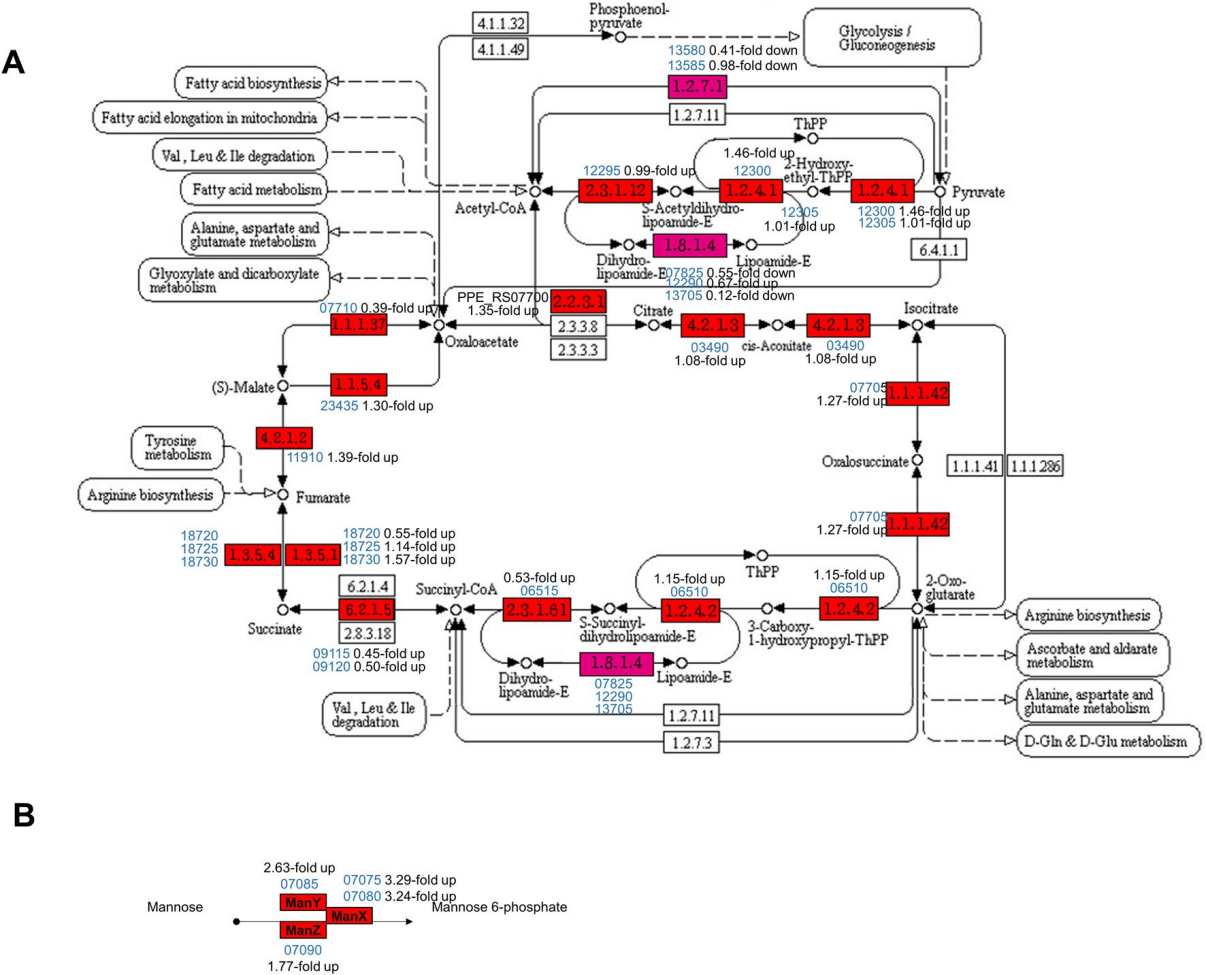
Upregulation of E681 genes involved in predicted carbohydrate metabolism. **(A)** Citrate cycle (TCA cycle) in F-type. The genes in E681 were marked red and all the genes were overexpressed in F-type. The graphic is adapted from the original KEGG pathway (ppy00020). **(B)** Mannose PTS encoded by *manXYZ.*

Genes related to carbohydrate metabolism were also upregulated in F-type, including those that group within the ‘Citrate cycle’ (ppy00020) and ‘Amino sugar and nucleotide sugar metabolism’ (ppy00520) KEGG pathways (Fig. 6A; Table S8). Further, 17 of the 23 genes in the ‘Amino sugar and nucleotide sugar metabolism’ pathways were upregulated in F-type compared to B-type. The expression of four *manXYZ* operon genes in the phosphotransferase system (PTS; ppy02060) was significantly increased (Fig. 6B).

### Environmental condition processing

Environmental condition processing is directly related to microbial survival, as it enables them to respond and adapt to fluctuating environmental conditions. Within this category, ‘two-component system’ (ppy02020) and ‘ABC transporter’ (ppy02010) pathways were upregulated in F-type (Tables S9 and S10). Of the 46 ‘two-component system’ genes, 32 were upregulated in F-type relative to B-type. With regards to genes involved in ABC transport, 42 of the 54 genes were overexpressed in F-type.

### Antimicrobial compound production

Interestingly, all antibiotic-related genes were downregulated in F-type (Table S11). In particular, the expression of *pnlA*, a peanilan gene, was significantly decreased (− 4.44-fold). The downregulation of antibiotic biosynthesis genes in F-type was consistent with the antifungal and antimicrobial activity shown in Figs. 2D–F. Further, the biosynthetic gene cluster of polymyxin, a clinically relevant antibiotic, includes five open reading frames, *pmxA*, *pmxB*, *pmxC*, *pmxD*, and *pmxE*^25^*.* Coincidentally, AbrB has been shown to inhibit the transcription of polymyxin biosynthetic genes^26^. This research supports the findings, as the expression of *abrB* (PPE_01425) was upregulated in F-type (1.39-fold), while expression of *pmsA/B/C/D/E* was downregulated. Thus, the downregulation of polymyxin genes in F-type may be mediated by *AbrB*.

## Discussion

Phenotypic variation has been observed in diverse bacteria, including many PGPR^27–29^, and appears to be associated with their adaptation to fluctuating environmental conditions. This variation affects root colonization and has been described in many PGPR. As phenotypic variation occurs widely in microorganisms, the mechanism underpinning the variation should be explored to help develop robust BCAs. Although *P. polymyxa* is a widely used BCA in agriculture and has been extensively studied^10,30,31^, no study has been conducted to explore this mechanism. This study is the first report to examine the molecular basis of phenotypic variation in *P. polymyxa.* It demonstrates that phenotypic variation is common in the species, as all 18 strains evaluated in this study exhibited phenotypic variation in liquid culture and on solid medium, on the seed surface and soil, and also in large scale culture needed to formulate biological control products. This variation likely reduces their ability to serve as a BCA.

Several strains of *P. polymyxa,* including E681, have many beneficial effects on crop plants and has been shown to produce a variety of antibiotics^25,32,33^. Many antagonistic bacteria secrete lytic enzymes that break down fungal cell walls, resulting in the suppression of fungal pathogens^34^. Additionally, proteases play a key role in the cell lysis process^35^. Proteases are represented by several *Bacillus* spp., including *B. amyloliquefaciens*, *B. licheniformis*, and *B. subtilis*^36,37^ which can suppress fungal plant pathogens. As described, the F-type, a naturally occurring variant, exhibits unique characteristics to the B-type, including attenuation of antimicrobial properties, biofilm formation, and plant growth-promotion. When compared with the F-type, B-type was more effective in promoting cucumber growth in terms of the length of sprouts from the seeds treated with the cell suspensions of B-type and F-type by dipping. This finding was further supported by the ability of B-type to produce higher levels of IAA compared to F-type. Similarly, a study performed by Heulin et al.^38^ reported an improvement in the yield of the wheat crops when IAA-producing rhizobacteria were applied to the plant roots. The remarkable difference between both types of E681 is the production of endospores. With the attenuation of the biological activities as a BCA, F-type does not form endospores, it can be a very critical issue for commercially available biocontrol agents^39^. Meanwhile, F-type was more competitive in motility than B-type. F-type had more flagella than B-type, and all flagella related RNAs were overexpressed in F-type, which correlated with increased swarming motility. Bacterial swarming describes the migration of cells across solid surfaces and is primarily powered by flagella. The swarming pattern was studied in several other *Paenibacillus* spp., such as *P. dendritiformis*, *P. aeruginosa*, and *B. subtilis* in which the event was regulated by multiple environmental factors^10^, including the production of surfactants on a solid surface^40,41^.

Our RNA sequencing data show that the sporulation is regulated at stage 0 in F-type. *B. subtilis* is considered as a model system for the study of sporulation^42^, based on this, the sporulation genes of E681 were classified into different sporulation stages^43,44^. The expression of genes on stage 0 was increased or decreased, but after stage II, all genes rapidly decreased in F-type. The master regulator for entry into sporulation is *Spo0A*, which has been shown to affect the expression of more than 100 genes^45–48^. In *B. subtilis*, sporulation begins with activation of the master transcription factor, *Spo0A*, which is initially transcribed from a sigma H (SigH)-dependent promoter^49,50^. The genetic regulation on stage 0 in F-type could relate to sigH, abrB, and kinases which are known to influence the expression of *Spo0A*. The factors involved in the decision-making process of *P. polymyxa* regarding sporulating remain unclear. Thus, further studies are needed to address the regulation of this phenomenon such as the initial steps or specific environmental cues involved. Although *spo0A* was upregulated in B-type compared to F-type, the expression levels of *spo0B* (PPE_03655) in B-type were like those in F-type, and the genes were not classified as differentially expressed. Moreover, *Kin1377* (PPE_01377) and *Kin1038* (PPE_01038) seem to be sporulation histidine kinases in E681 and their homologs in *B. subtilis* function in phosphorylation of *spo0A*^24^. Further, two global regulators, *abrB* and *sigH* (σ^H^), affect the regulation of *spo0A* in *B. subtilis*^48^. However, the regulation of these specific kinases and AbrB remains to be determined. It is, therefore, necessary to expand the study of phenotypic variation regulation to additional species. On the other hand, the upregulated gene in F-type was assigned to various cellular processes and functions, such as motility, energy production, and transportation systems. It revealed that genes involved in ‘flagella assembly’ and ‘environmental information processing’, particularly ATP-binding cassette (ABC) transporters were upregulated in F-type relative to B-type, whereas genes involved in ‘sporulation’ and ‘production of antibiotics’ were downregulated. Among transport systems, the Opp system was classified as belonging to the family of ABC transporters, which hydrolyze ATP to drive transport^51^. Similar results were shown in RNA-seq analysis using E681. ‘Transport’ was the most abundant subcategory identified as differentially enriched among gene ontology (GO). Enzymes involved in amino acid metabolism and transport were also upregulated, including oligopeptide-binding protein (OPPA), aminopeptidase (AP), and butanediol dehydrogenase (BDH). These results suggest that environmental stress increased the energy demand of the variant, which further increased mannose PTS activity due to insufficient glucose supply. Thus, the increased expression of ABC transporters in F-type is likely important to overcoming harsh environments.

We suggest that the bacteria can surrender their endospore-forming properties in unfavorable conditions. Alternatively, they may choose to escape from the harsh conditions by forming additional flagella, thereby reducing the need to produce biofilms or antibiotics. Notably, biofilm formation and antibiotic production were also reduced in F-type relative to B-type. The motility is mediated via downregulation of sporulation in stage 0, thereby, reducing unnecessary energy consumption and allowing for increased production of flagella, and other metabolic pathways, as well as an improved capacity to process environmental information. Very advanced research would be required to confirm this hypothesis.

One of the significant findings is that growth temperature is an important factor determining the degree of phenotypic variation. Though phenotypic variation helps bacteria to develop resistance for surviving under harsh environments^52^, this variation makes the bacteria less desirable as a BCA. This problem can be overcome by altering the growth temperature during mass production. It had not been reported previously that the occurrence of phenotypic variation caused by temperature. Our data show that the lower the culture temperature, the slower the time when the variant began to appear. When cultured at 37 °C, the first time the variant appeared was 2nd day, but when cultured at 24 °C, it was on the 6th day. When cultured at 20 °C and 18 °C, no variants appeared until the 7th day, the last day of observation, and the bacterial population reached 10^8^ cfu/mL. What is more powerful than controlling phenotypic variation is to select strains that are less likely to occur in the process of screening biocontrol agents. In case, when the biological product is made by exploiting a large yield of endospores upon culturing at a low temperature, if used in a summer farm, the variation might occur in the soil. The method we used was to observe whether the colonies have a uniform or various shape when spreading on a solid medium after liquid culture at 28 °C for 4 days. In this way, it was found that 2 out of 18 strains of *P. polymyxa* showed little variation. To get a good effect, there is also a way to adjust the timing of application to the farm. If a BCA is developed using a strain that does not easily change even at a high temperature, it may be used in a greenhouse or on a farm in summer. However, if the strain is prone to variation, it may be desirable to apply it when the soil temperature is below 24 °C for effective biocontrol. Phenotypic variation is a very critical issue in producing biocontrol products. Our study offers a simple solution that can be easily incorporated in large-scale BCA production helping increase the use of biocontrol.

## Materials and methods

### Strains and culture conditions

A total of 18 strains of *P. polymyxa* were used in this study (Fig. 3C; Table 1). Of these, M125 and six ginseng brown rot (GBR) strains have been reported to promote plant growth. The remaining 11 strains had not been previously isolated and were identified by our research group. *P. polymyxa* E681 was used throughout the study and is a spontaneous rifampicin mutant. To study typical bacterial growth, the wild type ‘B’ bacterial cells were cultured for 24 h in tryptic soy broth (TSB), followed by subculturing in fresh TSB and incubated for 4 days at 28 °C or 7 days at different temperatures (37, 30, 28, 24, 20, and 18 °C) under shaking conditions (180 rpm). The bacterial population thus cultured was recorded by plating serial dilutions on tryptic soy agar (TSA) plates. The total colony forming units (CFU)/mL and phenotypic variants were recorded at 24 h intervals.

### Phenotypic variation in vivo

To study phenotypic variation of E681 in seeds, surface-sterilized cucumber seeds were soaked in B-type cell suspensions (1 × 10^6^ cfu/mL) for 20 min, and air-dried for 3 h. Seeds were then placed on Petri-plates containing Whatman no. 1 filter paper under moisture conditions. To study phenotypic variation of E681 in soil, sterilized soil was mixed with B-type cell suspensions (1 × 10^6^ cfu/mL) and the soil was subsequently dried. Falcon tubes (50 mL capacity) were then filled with the bacteria-soil mixture and one surface-sterilized cucumber seed was planted per tube. SDW served as the non-treated control. Fifteen tubes were used for each treatment. All the samples were incubated at 28 °C for 11 days, at which point the length of the cucumber seedlings was measured, and the hypocotyl and root of the seedlings were cut, ground, and spread onto TSA plates for detecting the phenotypic variation by observing colony morphology. This experiment was performed two times.

### Transmission electron microscopy (TEM) and scanning electron microscopy (SEM) imaging

The protocols used for TEM and SEM analyses were described in the SI.

### Assessment of swarming motility, in vitro antagonism, and protease activity

The methods used for determining swarming motility, in vitro antagonism, and protease activity are described detailed in the SI.

### Siderophore production and biofilm formation

A detailed methods for measuring siderophore production and biofilm formation are describe in the SI.

### Quantification of IAA, and evaluation of plant growth-promoting effect on cucumbers

The experimental procedures used to determine IAA production, seed germination, PGP effect on cucumber seedlings are described in detail in the SI.

### RNA isolation and RNA-seq library preparation

Total RNA was isolated from cultures of B- and F-type cells grown in TSA for 48 h using the RNeasy Mini Kit with on-column DNase I treatment, according to the manufacturer’s instructions (Qiagen Inc., Hilden, Germany). RNA-seq libraries were prepared as described in Poulsen and Vinther^53^.

### RNA-seq analysis

After the sequencing of enriched mRNA, raw reads were mapped onto the E681 reference genome, using the program BWA. The E681 genome sequence was accessed through the NCBI genome database (https://www.ncbi.nlm.nih.gov/genome/; Accession No. NC_014483.2). In this study, we analyzed 4796 *P. polymyxa* E681 annotated genes. The resulting sequence alignment map (SAM) files were converted to binary format BAM files and then sorted by chromosomal coordinates using the program SAMtools. The number of mapped reads for each annotated gene was determined using the Bam2readcount function. The relative transcript abundance was measured in reads per kilobase of exon per million mapped sequence reads (RPKM). The log_2_ ratios of the RPKM values were used to identify differentially expressed genes (DEGs) and significance was set at *p* ≤ 0.01. Subsequently, DEGs were identified using the DEGseq package in R (https://www.r-project.org)^54^. The data from this analysis were deposited in the NCBI Gene Expression Omnibus (GEO) database and are accessible through GEO series GenBank accession no. GSE93062.

### Statistical analysis

Data were subjected to analysis of variance (ANOVA) using JMP software (SAS Institute Inc., Cary, NC, USA). The significance of B- and F-treated plant growth parameters was determined by the magnitude of the F value at *p* = 0.05. When a significant F value was obtained for treatments, the separation of the means was accomplished using Fisher's protected least significant difference (LSD) at *p* = 0.05.

## Supplementary information

Supplementary Information.
