# Supplementary material for: Molecular changes associated with spontaneous phenotypic variation of Paenibacillus polymyxa, a commonly used biocontrol agent, and temperature-dependent control of variation
Source: Sci Rep. 2020 Oct 6;10:16586. doi: 10.1038/s41598-020-73716-7 (PMC7538429; doi:10.1038/s41598-020-73716-7)
Supplement: Supplementary file 1 — Supplementary Information. [file 41598_2020_73716_MOESM1_ESM.docx]

**Molecular changes associated with spontaneous phenotypic variation of *Paenibacillus polymyxa*, a commonly used biocontrol agent, and temperature-dependent control of variation**

**Running title:** Phenotypic variation of *Paenibacillus polymyxa* and its control during mass production

Younmi Lee^1, 2^, Young Soo Kim^1^, Kotnala Balaraju^2^, Young-Su Seo^3^, Jungwook Park^3^, Choong-Min Ryu^4,5^, Seung-Hwan Park^4,5^, Jihyun F. Kim^6, 7^, Seogchan Kang^8^, Yongho Jeon^1^*

^1^Department of Plant Medicals, Andong National University, Andong 36729, Republic of Korea

^2^Agricultural Science & Technology Research Institute, Andong National University, Andong, 36729, Republic of Korea

^3^Department of Microbiology, Pusan National University, Pusan 46241, Republic of Korea

^4^Infectious Disease Research Centre, KRIBB, Daejeon 34141, Republic of Korea

^5^Department of Biosystems and Bioengineering, KRIBB School of Biotechnology, Korea University of Science and Technology, Daejeon 34141, Republic of Korea

^6^Department of Systems Biology, Division of Life Sciences, and Institute for Life Science and Biotechnology, Yonsei University, Seoul 03722, Republic of Korea

^7^Strategic Initiative for Microbiomes in Agriculture and Food (iMAF), Yonsei University, Seoul 03722, Republic of Korea

^8^Department of Plant Pathology & Environmental Microbiology, Pennsylvania State University, University Park, PA 16802, USA

*Corresponding author: Yongho Jeon

E-mail: [yongbac@andong.ac.kr](mailto:yongbac@andong.ac.kr)

Tel: +82-54-820-5507

Fax: +82-54-820-6320

**Supporting information (SI)**

**Materials and Methods**

**Mass culture.** To commercialize this bacterium for use in the field conditions, the bacterial culture (1.5 L) was inoculated into 250 L capacity fermenter (KoBioTech Co., Ltd., Incheon, Korea) containing industrial medium (Glucose 5.45g, MSG 3.25g, yeast extract 0.67g, KH_2_PO_4_ 0.27g, CaCl_2_ 0.27g, FeSO_4_ 0.005g, MgSO_4_ 0.08g, MnSO_4_ 0.005g, CuSO_4_ 0.003g, ZnSO_4_ 0.003g per liter), and incubated for 7 days at 30 ℃ with 80 rpm, and air pressure 50 L/min. The seed culture (inoculum) was prepared by culturing the bacterial cells in 4 L capacity conical flask containing industrial medium and incubating for 48 h at 30 ℃ with 150 rpm.

**Transmission electron microscopy (TEM) and scanning electron microscopy (SEM) imaging.** TEM was used to observe the morphological changes in both B- and F-types over 3 days. Samples for TEM were prepared and the images were captured as per the method described by Jeon *et al*^1^. Morphological and biochemical properties were identified, evaluated, and compared, as described in Bergey’s Manual of Systematic Bacteriology^2^. For SEM, the images of colonized root tips of cucumbers were captured as per the protocol described by Santos *et al*^3^.

**Assessment of swarming motility.** To assess for motility, 5 µl of B-type or F-type broth culture (1×10^6^ cfu/mL) was added dropwise on 1% agar TSA plates^4^. The diameters of the swarming zones were measured after incubation at 28 °C for 48 h.

**Siderophore production and biofilm formation.** Siderophore production was determined following the method of Schwyn and Neilands^5^. Briefly, a 6 μl sample of the 24-h-old bacterial cultures were spotted on CAS (chrome-azurol S) agar plates. The diameter of the orange halos was measured after the incubation of the plates at 28 °C for 48 h. The ability of the bacteria to form a biofilm on abiotic surfaces was evaluated using a method described previously by Ferrieres and Clarke^6^. Briefly, B-type and F-type colonies were cultured in TSB for 24 h, diluted 100-fold with TSB, and loaded into 96-well polystyrene plates (200 µl/well). TSB alone was used as a control. After incubating the cultures at 28 °C for 72 h without shaking, the cultures were carefully withdrawn with a pipette and the plates were washed twice with water. Each well received 200 µl of 1% (w/v) crystal violet, and the plates were allowed to stand for 15 min at room temperature. The solution was then withdrawn with a pipette, and the plates were carefully washed with water. Dye that had bound to the cells adsorbed on the walls of the wells was dissolved in acetone–ethanol (20:80; 200 µl/well). Biofilm-forming ability was evaluated by the intensity of the color of the solution. For the plates, the absorbance (OD at 595 nm) was read on a Multiskan Ascent analyzer (Thermo Fisher, Vantaa, Finland).

***In vitro* antagonism and protease activity.** The antagonistic properties of B- and F-types were tested *in vitro* against plant pathogenic fungi, including, *Rhizoctonia solani, Colletotrichum gloeosporioides,* and *Cylindrocarpon destructans*, and against *Escherichia coli* DH5α. All the isolates were provided by the Korean Agricultural Culture Collection (KACC), Suwon, Korea. Mycelial plugs were applied to the center of the potato dextrose agar (PDA) plate that was covered in a bacterial suspension. To test for antibacterial properties, *E. coli* DH5α was spread uniformly onto a TSA plate and 10 µl of B-type or F-type in TSB (1×10^6^ cfu/mL) was dropped onto a 5 mm sterile disk or directly onto the agar. Plates were incubated for 7 days at 28 °C and the antifungal/antibacterial activity was defined in terms of the zone of inhibition size. To test for protease activity *in vitro*, B-type and F-type cell suspensions were plated on TSA supplemented with 1% (w/v) skimmed milk. The plates were incubated for 7 days at 28 °C and zones of the clearing were indicative of enzyme activity.

**Determination of IAA quantification.** Indole-3-acetic acid (IAA) quantification assay was performed using the method described by Meza *et al*^7^. For the quantitative determination of IAA, the colorimetric Salkowski’s assay was performed^8^. IAA was quantified by integrating areas under peaks with authentic IAA (Sigma) as the standard.

**Plant growth-promoting effect on cucumbers.** Surface-sterilized cucumber (*Cucumis sativus* L. var. Eunsung-backdadagi) seeds were used for the growth-promoting assay. The surface-sterilized seeds were dipped in B-type or F-type cell suspensions (1 × 10^6^ cfu/mL) or SDW (control) for 20 min under shaking conditions at 150 rpm, and then air-dried. The seeds were transferred between the paper layers and kept in sealed plastic bags. The root lengths of germinated seedlings were measured 6 days after incubation at 20 ℃.

**References**

1. Jeon, Y. H., Kim, S. G., Hwang, I. & Kim, Y. H. Effects of initial inoculation density of *Paenibacillus polymyxa* on colony formation and starch-hydrolytic activity in relation to root rot in ginseng. *J Appl Microbiol* **109,** 461–470 (2010).
2. Debois, D. *et al*. Bergey’s Manual of Systematic Bacteriology Vol. 3 (eds P Vos *et al.*) (Springer, New York, 2009).
3. Santos F, *et al*. A novel interaction between plant-beneficial rhizobacteria and roots: colonization induces corn resistance against the root herbivore *Diabrotica speciosa*. *PLoS One* **9,** e113280 (2014).
4. Tremblay, J., Richardson, A., Lepine, F. & Deziel, E. Self-produced extracellular stimuli modulate the *Pseudomonas aeruginosa* swarming motility behaviour. *Envi Microbiol* **9,** 2622–2630 (2007).
5. Schwyn, B. & Neilands, J. B. Universal chemical assay for the detection and determination of siderophores. *Anal Biochem* **160,** 47–56 (1987).
6. Ferrieres, L & Clarke, D. The RcsC sensor kinase is required for normal biofilm formation in *Escherichia coli* K12 and controls the expression of a regulon in response to growth on a solid surface. *Mol Microbiol* **50,** 1665–1682 (2003).
7. Meza, B., de-Bashan, L. E. & Bashan, Y. Involvement of indole-3-acetic acid produced by *Azospirillum brasilense* in accumulating intracellular ammonium in *Chlorella vulgaris*. *Res Microbiol* **166,** 72–83 (2015).
8. Gordon, S. A. & Weber, R. P. Colorimetric estimation of indoleacetic acid. *Plant Physiol* **26,** 192-195 (1951).

# Supplementary tables

## **Table S1** Transcriptome sequencing results for B and F types of E681

| **Sample** | **Direction** | **Raw** | **Filtering** | **Matching** | **Mapping** |
| --- | --- | --- | --- | --- | --- |
| B | R1 | 80 090 435 | 74 092 479 | 66 735 650 | 32 069 380 |
| B | R2 | 80 090 435 | 70 050 839 | 66 735 650 |  |
| F | R1 | 69 408 784 | 65 164 819 | 59 801 781 | 55 575 382 |
| F | R2 | 69 408 784 | 62 026 047 | 59 801 781 |  |

## **Table S2** Gene Ontology (GO) functional annotation of differentially expressed genes (DEG).

| **Class** | **GO ID** | **GO** | **# of genes** | ***p*-value** |
| --- | --- | --- | --- | --- |
| **biological_process** | GO:0071973 | bacterial-type flagellum-dependent cell motility | 15 | 0.000 |
|  | GO:0006189 | 'de novo' IMP biosynthetic process | 13 | 0.000 |
|  | GO:0030435 | sporulation resulting in formation of a cellular spore | 10 | 0.000 |
|  | GO:0044780 | bacterial-type flagellum assembly | 8 | 0.000 |
|  | GO:0006935 | chemotaxis | 12 | 0.000 |
|  | GO:0008643 | carbohydrate transport | 22 | 0.000 |
|  | GO:0006810 | transport | 29 | 0.001 |
|  | GO:0006629 | lipid metabolic process | 6 | 0.003 |
|  | GO:0030436 | asexual sporulation | 4 | 0.007 |
|  | GO:0044781 | bacterial-type flagellum organization | 3 | 0.008 |
|  | GO:0006352 | DNA-templated transcription, initiation | 10 | 0.010 |
|  | GO:0044205 | 'de novo' UMP biosynthetic process | 5 | 0.011 |
|  | GO:0006099 | tricarboxylic acid cycle | 6 | 0.021 |
|  | GO:0007059 | chromosome segregation | 2 | 0.042 |
|  | GO:0009113 | purine nucleobase biosynthetic process | 2 | 0.042 |
|  | GO:0042882 | L-arabinose transport | 2 | 0.042 |
| **cellular_component** | GO:0009425 | bacterial-type flagellum basal body | 7 | 0.000 |
|  | GO:0016021 | integral component of membrane | 250 | 0.001 |
|  | GO:0009288 | bacterial-type flagellum | 3 | 0.008 |
|  | GO:0009424 | bacterial-type flagellum hook | 3 | 0.008 |
|  | GO:0005576 | extracellular region | 5 | 0.035 |
|  | GO:0005886 | plasma membrane | 59 | 0.041 |
|  | GO:0042601 | endospore-forming forespore | 2 | 0.042 |
| **molecular_function** | GO:0003774 | motor activity | 6 | 0.000 |
|  | GO:0005198 | structural molecule activity | 5 | 0.000 |
|  | GO:0004553 | hydrolase activity, hydrolyzing O-glycosyl compounds | 11 | 0.002 |
|  | GO:0016740 | transferase activity | 24 | 0.008 |
|  | GO:0004642 | phosphoribosylformylglycinamidine synthase activity | 3 | 0.008 |
|  | GO:0016987 | sigma factor activity | 10 | 0.014 |
|  | GO:0016829 | lyase activity | 11 | 0.017 |
|  | GO:0016757 | transferase activity, transferring glycosyl groups | 7 | 0.020 |
|  | GO:0008745 | N-acetylmuramoyl-L-alanine amidase activity | 4 | 0.036 |
|  | GO:0004088 | carbamoyl-phosphate synthase (glutamine-hydrolyzing) activity | 2 | 0.042 |
|  | GO:0004644 | phosphoribosylglycinamide formyltransferase activity | 2 | 0.042 |
|  | GO:0004739 | pyruvate dehydrogenase (acetyl-transferring) activity | 2 | 0.042 |
|  | GO:0005509 | calcium ion binding | 2 | 0.042 |
|  | GO:0008514 | organic anion transmembrane transporter activity | 2 | 0.042 |
|  | GO:0008565 | protein transporter activity | 2 | 0.042 |
|  | GO:0015612 | L-arabinose-importing ATPase activity | 2 | 0.042 |
|  | GO:0043365 | [formate-C-acetyltransferase]-activating enzyme activity | 2 | 0.042 |
|  | GO:0045735 | nutrient reservoir activity | 2 | 0.042 |
|  | GO:0047371 | butyrate-acetoacetate CoA-transferase activity | 2 | 0.042 |
|  | GO:0051539 | 4 iron, 4 sulfur cluster binding | 13 | 0.049 |

## **Table S3** Summary of KEGG pathways associated with ≥ 2-fold DEGs

| **ID** | **Pathway** | **p-value** |
| --- | --- | --- |
| ppy02040 | Flagellar assembly | 0 |
| ppy02030 | Bacterial chemotaxis | 3.35E-11 |
| ppy00020 | Citrate cycle (TCA cycle) | 0.000121325 |
| ppy00520 | Amino sugar and nucleotide sugar metabolism | 0.000489019 |
| ppy02020 | Two-component system | 0.002995599 |
| ppy02010 | ABC transporters | 0.003554139 |
| ppy00670 | One carbon pool by folate | 0.027934135 |
| ppy00650 | Butanoate metabolism | 2.49E-06 |
| ppy00071 | Fatty acid degradation | 8.49E-05 |
| ppy00625 | Chloroalkane and chloroalkene degradation | 0.000241879 |
| ppy00626 | Naphthalene degradation | 0.000279292 |
| ppy00350 | Tyrosine metabolism | 0.001063487 |
| ppy00640 | Propanoate metabolism | 0.002539041 |
| ppy01100 | Metabolic pathways | 0.004282199 |
| ppy00072 | Synthesis and degradation of ketone bodies | 0.004315507 |
| ppy01220 | Degradation of aromatic compounds | 0.00482077 |
| ppy00310 | Lysine degradation | 0.008034461 |
| ppy03450 | Non-homologous end-joining | 0.012387936 |
| ppy00440 | Phosphonate and phosphinate metabolism | 0.017497167 |
| ppy01120 | Microbial metabolism in diverse environments | 0.020762284 |
| ppy00620 | Pyruvate metabolism | 0.03485157 |
| ppy00627 | Aminobenzoate degradation | 0.037833094 |

## **Table S4** Expression of all the genes constituting GO pathways associated with sporulation

| GO id | GO | Description | Locus_tag | B | F | log2  Fold change | p-value |
| --- | --- | --- | --- | --- | --- | --- | --- |
| GO:  0030435 | sporulation resulting in formation of a cellular spore | RNA polymerase sigma factor SigD | PPE_RS09310 | 113.19 | 287.67 | 1.45 | 0.000 |
|  |  | chemotaxis protein CheY | PPE_RS13740 | 1371.56 | 538.73 | -1.24 | 0.000 |
|  |  | septation protein spoVG | PPE_RS00180 | 5453.67 | 1342.65 | -1.92 | 0.000 |
|  |  | DNA-directed RNA polymerase sigma-70 factor | PPE_RS15800 | 43.18 | 10.25 | -1.97 | 0.000 |
|  |  | anti-sigma F factor antagonist | PPE_RS13625 | 3205.97 | 182.99 | -4.03 | 0.000 |
|  |  | DNA-directed RNA polymerase sigma-70 factor | PPE_RS15115 | 694.61 | 33.14 | -4.28 | 0.000 |
|  |  | anti-sigma F factor | PPE_RS13620 | 4352.00 | 180.80 | -4.48 | 0.000 |
|  |  | DNA-directed RNA polymerase sigma-70 factor | PPE_RS15120 | 569.22 | 17.32 | -4.93 | 0.000 |
|  |  | stage II sporulation protein D | PPE_RS21545 | 349.81 | 6.22 | -5.71 | 0.000 |
|  |  | stage IV sporulation protein A | PPE_RS13410 | 4635.11 | 16.50 | -8.03 | 0.000 |
| GO:  0030436 | asexual sporulation | acid-soluble spore protein H | PPE_RS20155 | 107.14 | 17.72 | -2.49 | 0.000 |
|  |  | spore protein | PPE_RS05655 | 63.06 | 10.20 | -2.52 | 0.000 |
|  |  | anti-sigma F factor | PPE_RS13620 | 4352.00 | 180.80 | -4.48 | 0.000 |
|  |  | peptidase | PPE_RS15125 | 1345.99 | 28.99 | -5.43 | 0.000 |
| GO:  0042601 | endospore-forming forespore | acid-soluble spore protein H | PPE_RS20155 | 107.14 | 17.72 | -2.49 | 0.000 |
|  |  | spore protein | PPE_RS05655 | 63.06 | 10.20 | -2.52 | 0.000 |

## **Table S5** Expression of all the genes related to sporulation stage 0

| **Description** | **locus_tag** | **old_locus** | **B** | **F** | **log2 Fold_change** | **p-value** | **Signature**  **(p-value < 0.01)** |
| --- | --- | --- | --- | --- | --- | --- | --- |
| soj | PPE_RS23930 | PPE_04944 | 87.16 | 150.23 | 0.89 | 0.000 | TRUE |
| spo0J | PPE_RS23925 | PPE_04943 | 145.30 | 214.04 | 0.66 | 0.000 | TRUE |
| sigH | PPE_RS20805 | PPE_04296 | 10353.91 | 4585.43 | -1.07 | 0.000 | TRUE |
| kin3851 | PPE_RS18645 | PPE_03851 | 60.22 | 24.67 | -1.18 | 0.000 | TRUE |
| spo0B | PPE_RS17680 | PPE_03655 | 163.52 | 182.67 | 0.26 | 0.088 | FALSE |
| spo0A | PPE_RS13740 | PPE_02831 | 1371.56 | 538.73 | -1.24 | 0.000 | TRUE |
| spo0M | PPE_RS13720 | PPE_02826 | 365.76 | 266.27 | -0.35 | 0.002 | TRUE |
| spo0F | PPE_RS00675 | PPE_00136 | 683.14 | 342.22 | -0.89 | 0.000 | TRUE |
| abrB | PPE_RS00125 | PPE_00024 | 377.37 | 561.12 | 0.68 | 0.000 | TRUE |
| abrB | PPE-RS06890 | PPE_01425 | 28.80 | 70.38 | 1.29 | 0.000 | TRUE |
| yaaT | PPE_RS00105 | PPE_00020 | 176.97 | 210.13 | 0.35 | 0.016 | FALSE |

## **Table S6** Expression of kinase genes

| **Description** | **locus_tag** | **old_locus** | **B** | **F** | **log2 Fold_change** | **p-value** | **Signature (p-value < 0.05)** |
| --- | --- | --- | --- | --- | --- | --- | --- |
| histidine kinase Kin3851 | PPE_RS18645 | PPE_03851 | 60.22 | 24.67 | -1.18 | 0.000 | TRUE |
| histidine kinase Kin1377 | PPE_RS06650 | PPE_01377 | 817.58 | 395.86 | -0.94 | 0.000 | TRUE |
| histidine kinase Kin1038 | PPE_RS05010 | PPE_01038 | 59.43 | 43.72 | -0.34 | 0.237 | FALSE |
| histidine kinase Kin689 | PPE_RS03360 | PPE_00689 | 57.49 | 81.47 | 0.61 | 0.014 | TRUE |
| histidine kinase Kin99 | PPE_RS00495 | PPE_00099 | 100.24 | 241.12 | 1.37 | 0.000 | TRUE |

## **Table S7** Expression of all the genes constituting bacterial chemotaxis (ppy02030) and Flagella assembly (ppy02040) of KEGG pathway

| **pathway** | **Description** | **locus_tag** | **log2 Fold_change** | **p-value** |
| --- | --- | --- | --- | --- |
| Bacterial chemotaxis (ppy02030) | chemotaxis protein | PPE_RS17265 | 1.57 | 0.002 |
|  | chemotaxis protein | PPE_RS13730 | 1.77 | 0.001 |
|  | chemotaxis protein | PPE_RS14495 | 1.07 | 0.001 |
|  | chemotaxis protein | PPE_RS02325 | 1.06 | 0.000 |
|  | LacI family transcriptional regulator | PPE_RS05065 | 1.53 | 0.000 |
|  | chemotaxis protein | PPE_RS17485 | 1.47 | 0.000 |
|  | chemotaxis protein | PPE_RS22075 | -2.01 | 0.000 |
|  | sugar ABC transporter substrate-binding protein | PPE_RS04180 | 2.06 | 0.000 |
|  | chemotaxis protein | PPE_RS16160 | 2.02 | 0.000 |
|  | chemotaxis protein CheD | PPE_RS09300 | 1.14 | 0.000 |
|  | chemotaxis protein CheY | PPE_RS10395 | 1.46 | 0.000 |
|  | chemotaxis protein | PPE_RS05835 | 2.02 | 0.000 |
|  | flagellar motor protein MotA | PPE_RS21065 | 1.48 | 0.000 |
|  | flagellar motor protein MotB | PPE_RS00505 | 1.51 | 0.000 |
|  | chemotaxis protein R | PPE_RS19490 | 1.05 | 0.000 |
|  | chemotaxis protein CheW | PPE_RS09290 | 1.43 | 0.000 |
|  | chemotaxis protein | PPE_RS18220 | 1.51 | 0.000 |
|  | flagellar motor protein MotA | PPE_RS00510 | 1.77 | 0.000 |
|  | two-component system protein-glutamate methylesterase response regulator | PPE_RS09280 | 2.10 | 0.000 |
|  | sugar ABC transporter substrate-binding protein | PPE_RS04165 | 1.91 | 0.000 |
|  | chemotaxis protein CheY | PPE_RS09235 | 1.39 | 0.000 |
|  | chemotaxis protein CheA | PPE_RS09285 | 1.99 | 0.000 |
|  | flagellar motor switch protein FliN | PPE_RS09230 | 2.56 | 0.000 |
|  | flagellar motor switch protein FliM | PPE_RS09225 | 2.42 | 0.000 |
|  | flagellar motor switch protein FliG | PPE_RS09170 | 3.31 | 0.000 |
| Flagellar assembly (ppy02040) | flagellar biosynthesis protein FlhB | PPE_RS09260 | 1.78 | 0.000 |
|  | flagellar biosynthesis protein FliQ | PPE_RS09250 | 2.01 | 0.000 |
|  | flagellar biosynthesis protein FliP | PPE_RS09245 | 1.62 | 0.000 |
|  | flagellar motor protein MotA | PPE_RS21065 | 1.48 | 0.000 |
|  | flagellar biosynthesis protein FliR | PPE_RS09255 | 2.06 | 0.000 |
|  | flagellar biosynthesis protein FlhA | PPE_RS09265 | 1.92 | 0.000 |
|  | flagellar protein | PPE_RS09240 | 1.53 | 0.000 |
|  | flagellar export chaperone FliS | PPE_RS21340 | 1.23 | 0.000 |
|  | flagellar motor protein MotB | PPE_RS00505 | 1.51 | 0.000 |
|  | flagellar motor protein MotA | PPE_RS00510 | 1.77 | 0.000 |
|  | flagellar basal body rod protein | PPE_RS21525 | 1.00 | 0.000 |
|  | flagellar hook protein FlgL | PPE_RS21370 | 1.82 | 0.000 |
|  | flagellar hook capping protein | PPE_RS09200 | 2.83 | 0.000 |
|  | flagellar hook protein FlgK | PPE_RS21375 | 2.39 | 0.000 |
|  | flagellar hook-length control protein | PPE_RS09195 | 2.57 | 0.000 |
|  | flagellar cap protein FliD | PPE_RS21345 | 2.15 | 0.000 |
|  | flagellar motor switch protein FliN | PPE_RS09230 | 2.56 | 0.000 |
|  | flagellar biosynthesis protein | PPE_RS09175 | 3.10 | 0.000 |
|  | flagellar motor switch protein FliM | PPE_RS09225 | 2.42 | 0.000 |
|  | flagellar basal body rod protein FlgG | PPE_RS09210 | 2.62 | 0.000 |
|  | flagellar motor switch protein FliG | PPE_RS09170 | 3.31 | 0.000 |
|  | hypothetical protein | PPE_RS09185 | 2.72 | 0.000 |
|  | ATP synthase | PPE_RS09180 | 3.05 | 0.000 |
|  | flagellar M-ring protein FliF | PPE_RS09165 | 2.96 | 0.000 |
|  | flagellar hook-basal body protein FliE | PPE_RS09160 | 3.26 | 0.000 |
|  | flagellin synthesis anti-sigma-D factor | PPE_RS21385 | 2.64 | 0.000 |
|  | flagellar basal body rod protein FlgC | PPE_RS09155 | 3.22 | 0.000 |
|  | flagellar biosynthesis protein FlgB | PPE_RS09150 | 3.62 | 0.000 |
|  | flagellin | PPE_RS21355 | 2.21 | 0.000 |

## **Table S8** Expression of all the genes associated with ‘amino sugar and nucleotide sugar metabolism (ppy00520)

| **Description** | **locus** | **B** | **F** | **log2 Fold change** | **p-value** |
| --- | --- | --- | --- | --- | --- |
| PTS mannose transporter subunit IID | PPE_01463 | 151.01 | 1376.68 | 3.29 | 0.000 |
| PTS fructose transporter subunit IIB | PPE_01464 | 225.31 | 1982.65 | 3.24 | 0.000 |
| PTS mannose transporter subunit IIC | PPE_01465 | 424.02 | 2439.99 | 2.63 | 0.000 |
| xylosidase | PPE_01656 | 102.06 | 448.43 | 2.24 | 0.000 |
| glucose-1-phosphate adenylyltransferase | PPE_02517 | 206.56 | 817.53 | 2.09 | 0.000 |
| PTS mannose transporter subunit IID | PPE_01466 | 877.49 | 2783.36 | 1.77 | 0.000 |
| flagellar modification protein B | PPE_04402 | 17.92 | 50.44 | 1.60 | 0.000 |
| arabinoxylan arabinofuranohydrolase | PPE_02273 | 395.47 | 1068.51 | 1.54 | 0.000 |
| alpha-N-arabinofuranosidase | PPE_00320 | 113.04 | 266.28 | 1.34 | 0.000 |
| galactokinase | PPE_01014 | 90.53 | 210.76 | 1.32 | 0.000 |
| glutamine amidotransferase | PPE_04214 | 1291.05 | 2864.92 | 1.25 | 0.000 |
| hypothetical protein | PPE_04401 | 41.64 | 89.39 | 1.21 | 0.000 |
| PTS trehalose transporter subunit IIBC | PPE_00251 | 401.31 | 854.59 | 1.20 | 0.000 |
| alpha-N-arabinofuranosidase | PPE_02888 | 39.43 | 83.75 | 1.19 | 0.000 |
| UDP-N-acetylglucosamine 2-epimerase | PPE_04400 | 42.29 | 88.14 | 1.16 | 0.000 |
| UDP-N-acetylglucosamine 1-carboxyvinyltransferase | PPE_00138 | 102.85 | 205.13 | 1.10 | 0.000 |
| UDP-glucose 6-dehydrogenase | PPE_01134 | 156.55 | 303.44 | 1.06 | 0.000 |
| UDP-N-acetylenolpyruvoylglucosamine reductase | PPE_03126 | 487.49 | 213.90 | -1.08 | 0.000 |
| nucleoside-diphosphate sugar epimerase | PPE_03826 | 22.35 | 4.68 | -2.15 | 0.001 |
| mannose-1-phosphate guanylyltransferase | PPE_02199 | 33.08 | 5.70 | -2.43 | 0.000 |
| UDP-N-acetyl-D-glucosamine dehydrogenase | PPE_03756 | 38.62 | 5.60 | -2.68 | 0.000 |
| UDP-glucose 4-epimerase | PPE_03815 | 52.37 | 6.96 | -2.81 | 0.000 |
| UDP-N-acetylglucosamine 2-epimerase | PPE_03829 | 33.24 | 4.05 | -2.93 | 0.000 |

## **Table S9** Expression of all the genes associated with ‘two-component system’ (ppy02020)

| **Description** | **old_locus** | **B** | **F** | **log2 Fold_change** | **p-value** |
| --- | --- | --- | --- | --- | --- |
| flagellar motor protein MotA | PPE_00102 | 80.33 | 254.63 | 1.77 | 0.000 |
| chemotaxis protein | PPE_00476 | 32.50 | 63.20 | 1.06 | 0.000 |
| histidine kinase | PPE_00721 | 43.61 | 93.28 | 1.20 | 0.000 |
| two-component system response regulator | PPE_00722 | 69.32 | 177.49 | 1.46 | 0.000 |
| fatty acid desaturase, Des | PPE_00885 | 213.87 | 448.59 | 1.17 | 0.000 |
| sensor histidine kinase, DesK | PPE_00886 | 43.72 | 89.87 | 1.14 | 0.000 |
| Transcriptional regulator, DesR | PPE_00887 | 29.73 | 53.44 | 0.95 | 0.003 |
| histidine kinase | PPE_01051 | 40.90 | 101.96 | 1.42 | 0.000 |
| two-component system response regulator | PPE_01052 | 38.56 | 110.39 | 1.62 | 0.000 |
| succinyl-CoA--3-ketoacid-CoA transferase | PPE_01172 | 130.61 | 4.82 | -4.66 | 0.000 |
| 3-oxoadipate CoA-transferase subunit A | PPE_01173 | 543.01 | 13.93 | -5.18 | 0.000 |
| acetyl-CoA acetyltransferase | PPE_01175 | 450.76 | 13.73 | -4.93 | 0.000 |
| chemotaxis protein | PPE_01208 | 21.10 | 79.59 | 2.02 | 0.000 |
| NAD-dependent malic enzyme (NAD-ME) | PPE_01305 | 381.04 | 929.96 | 1.39 | 0.000 |
| chemotaxis protein CheY | PPE_01454 | 70.28 | 17.41 | -1.91 | 0.000 |
| chemotaxis protein CheY | PPE_01652 | 30.91 | 76.13 | 1.41 | 0.000 |
| chemotaxis protein CheY | PPE_01897 | 240.60 | 585.04 | 1.39 | 0.000 |
| two-component system protein-glutamate methylesterase response regulator | PPE_01906 | 54.10 | 216.32 | 2.10 | 0.000 |
| chemotaxis protein CheA | PPE_01907 | 99.62 | 367.96 | 1.99 | 0.000 |
| chemotaxis protein CheW | PPE_01908 | 103.94 | 260.48 | 1.43 | 0.000 |
| RNA polymerase sigma factor SigD | PPE_01912 | 113.19 | 287.67 | 1.45 | 0.000 |
| chemotaxis protein CheY | PPE_02134 | 41.23 | 105.19 | 1.46 | 0.000 |
| chemotaxis protein | PPE_02829 | 7.52 | 23.86 | 1.77 | 0.001 |
| chemotaxis protein CheY | PPE_02831 | 1371.56 | 538.73 | -1.24 | 0.000 |
| chemotaxis protein | PPE_02987 | 27.75 | 54.21 | 1.07 | 0.001 |
| NAD-dependent malic enzyme 4 (NAD-ME 4) | PPE_03209 | 31.16 | 6.61 | -2.13 | 0.000 |
| L-malate permease | PPE_03210 | 51.99 | 12.48 | -1.95 | 0.000 |
| chemotaxis protein | PPE_03338 | 14.14 | 53.44 | 2.02 | 0.000 |
| histidine kinase | PPE_03457 | 32.86 | 79.99 | 1.39 | 0.000 |
| chemotaxis protein | PPE_03569 | 9.62 | 26.63 | 1.57 | 0.002 |
| 2-(5''-triphosphoribosyl)-3'-dephospho-CoA synthase | PPE_03593 | 17.35 | 5.96 | -1.44 | 0.025 |
| DNA-binding protein | PPE_03594 | 42.61 | 18.42 | -1.10 | 0.004 |
| chemotaxis protein | PPE_03616 | 19.72 | 50.69 | 1.47 | 0.000 |
| malate dehydrogenase | PPE_03731 | 25.97 | 4.20 | -2.52 | 0.000 |
| chemotaxis protein | PPE_03762 | 113.56 | 299.80 | 1.51 | 0.000 |
| two-component system sensor histidine kinase | PPE_03851 | 60.22 | 24.67 | -1.18 | 0.000 |
| chemotaxis protein R | PPE_04022 | 214.19 | 413.15 | 1.05 | 0.000 |
| cytochrome D ubiquinol oxidase subunit II | PPE_04044 | 88.47 | 6.49 | -3.66 | 0.000 |
| cytochrome D ubiquinol oxidase subunit I | PPE_04045 | 74.73 | 5.02 | -3.79 | 0.000 |
| two-component system response regulator | PPE_04086 | 17.74 | 50.11 | 1.60 | 0.000 |
| histidine kinase | PPE_04087 | 25.14 | 83.19 | 1.83 | 0.000 |
| flagellar motor protein MotA | PPE_04350 | 48.36 | 125.22 | 1.48 | 0.000 |
| flagellin | PPE_04409 | 3920.25 | 16847.96 | 2.21 | 0.000 |
| chemotaxis protein | PPE_04558 | 47.47 | 10.93 | -2.01 | 0.000 |
| two-component system response regulator | PPE_04713 | 20.26 | 69.31 | 1.88 | 0.000 |
| bacitracin ABC transporter ATP-binding protein | PPE_04799 | 32.00 | 80.90 | 1.44 | 0.000 |

## **Table S10** The genes in ‘ABC transporter’ (ppy02010) of KEGG pathway

| **Gene ID** | **Description** | **RPKM** | | **log2 Fold change** |
| --- | --- | --- | --- | --- |
|  |  | **‘B’** | **‘F’** |  |
| PPE_01654 | sugar ABC transporter permease | 111.2 | 870.0 | 3.07 |
| PPE_00517 | sugar ABC transporter permease | 333.6 | 2166.4 | 2.80 |
| PPE_00516 | ABC transporter permease | 304.9 | 1879.7 | 2.73 |
| PPE_01653 | sugar ABC transporter substrate-binding protein | 344.8 | 1809.2 | 2.50 |
| PPE_00642 | sugar ABC transporter substrate-binding protein | 79.1 | 409.6 | 2.48 |
| PPE_01655 | sugar ABC transporter permease | 167.0 | 723.6 | 2.22 |
| PPE_03455 | ABC transporter substrate-binding protein | 19.8 | 83.3 | 2.18 |
| PPE_04079 | sugar ABC transporter permease | 5.5 | 23.1 | 2.17 |
| PPE_04159 | sugar ABC transporter permease | 403.7 | 1606.8 | 2.10 |
| PPE_00860 | sugar ABC transporter substrate-binding protein | 12.2 | 47.3 | 2.06 |
| PPE_00643 | ABC transporter permease | 75.9 | 281.5 | 2.00 |
| PPE_00857 | sugar ABC transporter substrate-binding protein | 81.0 | 283.2 | 1.91 |
| PPE_04641 | ABC transporter substrate-binding protein | 27.8 | 96.2 | 1.89 |
| PPE_04467 | ABC transporter permease | 18.2 | 59.5 | 1.81 |
| PPE_02056 | ABC transporter permease | 23.5 | 73.2 | 1.75 |
| PPE_04161 | ABC transporter substrate-binding protein | 706.9 | 2197.7 | 1.74 |
| PPE_00862 | ABC transporter permease | 9.9 | 29.7 | 1.69 |
| PPE_00861 | sugar ABC transporter ATP-binding protein | 14.8 | 44.1 | 1.68 |
| PPE_01780 | ABC transporter substrate-binding protein | 109.2 | 318.7 | 1.65 |
| PPE_04078 | sugar ABC transporter permease | 15.4 | 43.2 | 1.59 |
| PPE_04160 | ABC transporter permease | 700.8 | 1933.8 | 1.57 |
| PPE_00644 | sugar ABC transporter permease | 59.4 | 160.2 | 1.54 |
| PPE_01050 | LacI family transcriptional regulator | 16.3 | 43.6 | 1.53 |
| PPE_04336 | ABC transporter ATP-binding protein | 45.2 | 114.4 | 1.44 |
| PPE_04799 | bacitracin ABC transporter ATP-binding protein | 32.0 | 80.9 | 1.44 |
| PPE_02388 | sugar ABC transporter permease | 17.9 | 44.8 | 1.43 |
| PPE_02055 | sugar ABC transporter permease | 19.4 | 48.2 | 1.42 |
| PPE_03454 | ABC transporter permease | 33.9 | 83.8 | 1.41 |
| PPE_00518 | ABC transporter substrate-binding protein | 876.2 | 2156.9 | 1.40 |
| PPE_04466 | sugar ABC transporter permease | 31.2 | 75.0 | 1.37 |
| PPE_04465 | ABC transporter substrate-binding protein | 15.8 | 34.8 | 1.24 |
| PPE_04753 | nickel ABC transporter permease | 12.6 | 27.5 | 1.23 |
| PPE_03091 | protein lplB | 28.8 | 59.6 | 1.15 |
| PPE_04640 | sugar ABC transporter permease | 127.9 | 261.5 | 1.14 |
| PPE_03092 | ABC transporter permease | 48.4 | 97.8 | 1.12 |
| PPE_04754 | nickel ABC transporter substrate-binding protein | 39.8 | 79.7 | 1.11 |
| PPE_04337 | teichoic acid ABC transporter permease | 37.6 | 75.3 | 1.11 |
| PPE_03699 | sugar ABC transporter substrate-binding protein | 26.7 | 52.4 | 1.08 |
| PPE_02485 | D-ribose transporter ATP-binding protein | 45.7 | 89.8 | 1.08 |
| PPE_04368 | cell division protein FtsE | 72.9 | 140.3 | 1.05 |
| PPE_00790 | arabinose-binding protein | 138.5 | 265.3 | 1.04 |
| PPE_03089 | sugar ABC transporter substrate-binding protein | 48.4 | 91.2 | 1.02 |
| PPE_00779 | iron-dicitrate ABC transporter permease | 129.6 | 56.8 | -1.08 |
| PPE_03390 | molybdenum ABC transporter substrate-binding protein | 35.8 | 14.7 | -1.18 |
| PPE_03389 | molybdenum ABC transporter permease | 20.6 | 8.3 | -1.21 |
| PPE_04082 | ATP-binding protein | 25.8 | 9.8 | -1.29 |
| PPE_00399 | glutamine ABC transporter permease | 31.5 | 10.1 | -1.54 |
| PPE_01198 | glycerol-3-phosphate ABC transporter permease | 20.8 | 6.3 | -1.63 |
| PPE_00398 | glutamine ABC transporter permease | 35.3 | 9.5 | -1.79 |
| PPE_01168 | multidrug ABC transporter | 246.6 | 58.4 | -1.97 |
| PPE_00400 | glutamine ABC transporter substrate-binding protein | 31.4 | 7.4 | -1.99 |
| PPE_01199 | ABC transporter permease | 34.7 | 7.0 | -2.20 |
| PPE_04708 | bacitracin ABC transporter ATP-binding protein | 1180.2 | 209.5 | -2.39 |
| PPE_00401 | amino acid ABC transporter ATPase | 58.1 | 5.6 | -3.27 |

**Table S11. Expression of antibiotic biosynthesis genes in B- and F-type cells.**

|  | **Description** | **symbol** | **locus** | **B** | **F** | **log2 Fold change** | **p-value** |
| --- | --- | --- | --- | --- | --- | --- | --- |
| Fusaricidin | non-ribosomal peptide synthetase | FusA | PPE_00083 | 352.67 | 205.63 | -0.67 | 0.000 |
| Polymyxin | non-ribosomal peptide synthetase | pmxA | PPE_04188 | 360.06 | 192.05 | -0.80 | 0.000 |
|  | non-ribosomal peptide synthetase | pmxB | PPE_04187 | 310.98 | 159.53 | -0.86 | 0.000 |
|  | multidrug ABC transporter permease | pmxC | PPE_04186 | 379.79 | 168.61 | -1.07 | 0.000 |
|  | ABC transporter ATP-binding protein | pmxD | PPE_04185 | 272.20 | 114.90 | -1.24 | 0.000 |
|  | non-ribosomal peptide synthetase | pmxE | PPE_04184 | 157.08 | 64.99 | -1.17 | 0.000 |
| Lantibiotics | ABC transporter ATP-binding protein | SpaT | PPE_01456 | 85.81 | 27.25 | -1.55 | 0.000 |
|  | lantibiotic biosynthesis protein | SpaB | PPE_01455 | 58.90 | 17.87 | -1.62 | 0.000 |
|  | lantibiotic biosynthesis protein | SpaC | PPE_01453 | 86.46 | 19.92 | -2.01 | 0.000 |
|  | lantibiotic biosynthesis protein | SpaC | PPE_01452 | 458.85 | 87.75 | -2.28 | 0.000 |
| Paenilan | subtilin lantibiotic | pnlA | PPE_04953 | 6825.48 | 293.07 | -4.44 | 0.000 |
| Phospho-glucomutase | phosphoglucomutase | pgm | PPE_04441 | 350.28 | 274.29 | -0.25 | 0.032 |

**Supplementary Figures**


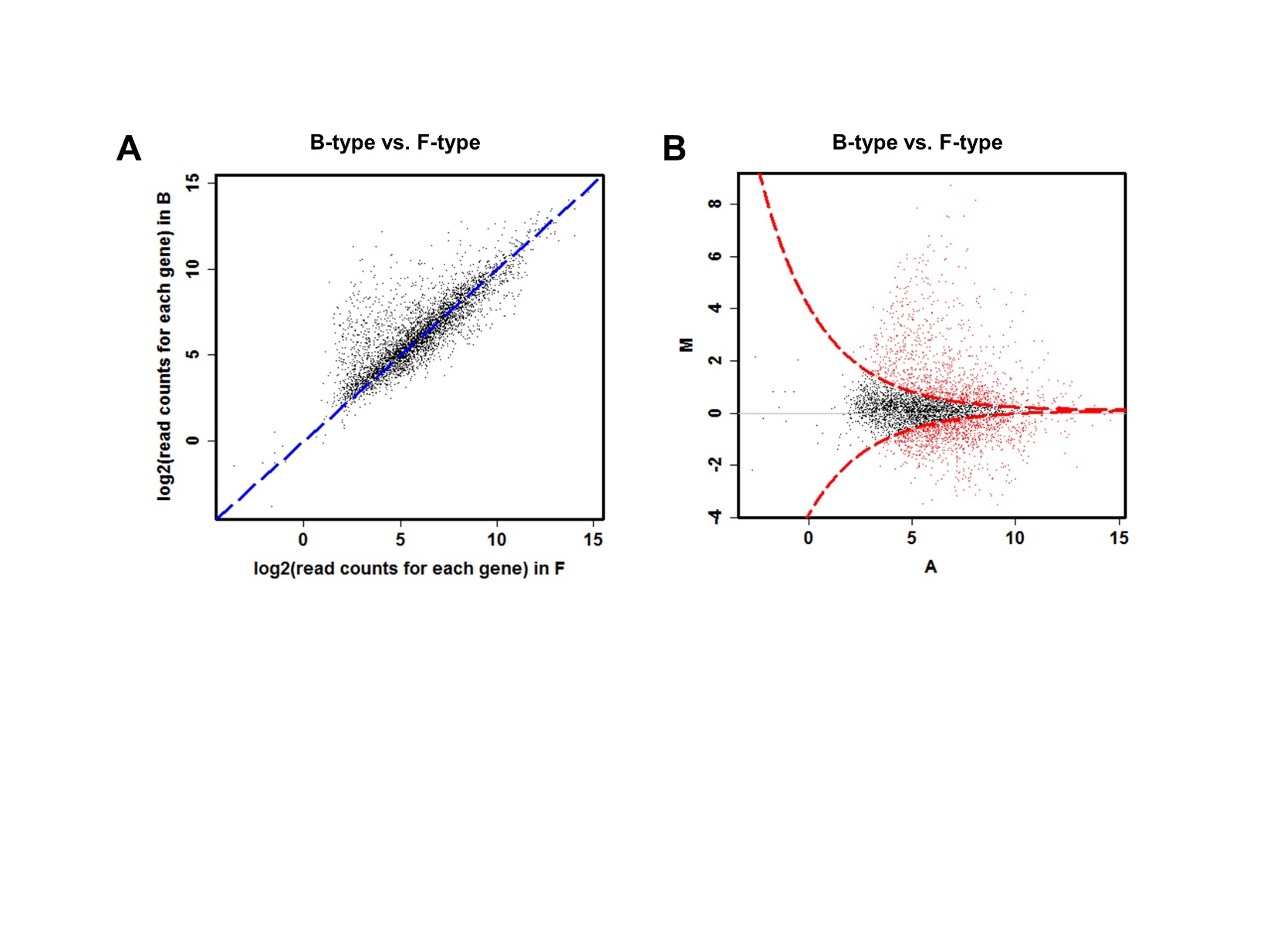


**Fig. S1.** Differentially expressed genes were represented as a scatterplot graph (**A**) and MA-plot graph (**B**) by the DEGseq package with the R language. Each point indicates 4,796 genes. In the scatterplot graph, it is possible to compare the expression level between two groups through the x-axis of read counts *in vitro* and y-axis of read counts. Red and black points of the MA-plot refer to those differentially and not differentially expressed genes, respectively. In this analysis, red points follow statistic criteria with a *p*-value ≤ 0.01 as determined by the MARS method.


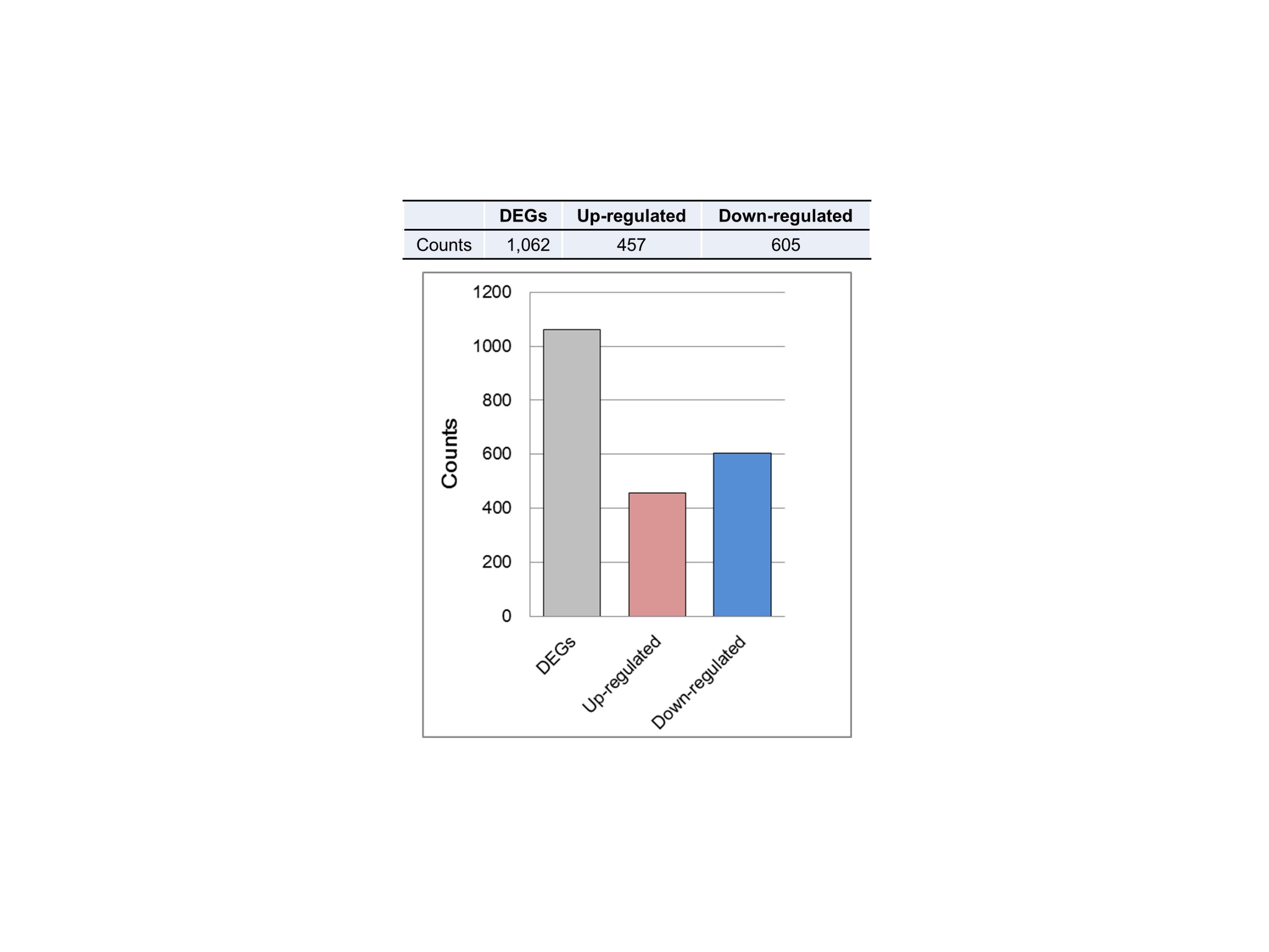


**Fig. S2.** Counts of DEGs. The number of upregulated and downregulated DEGs in the F-type compared with the B-type.


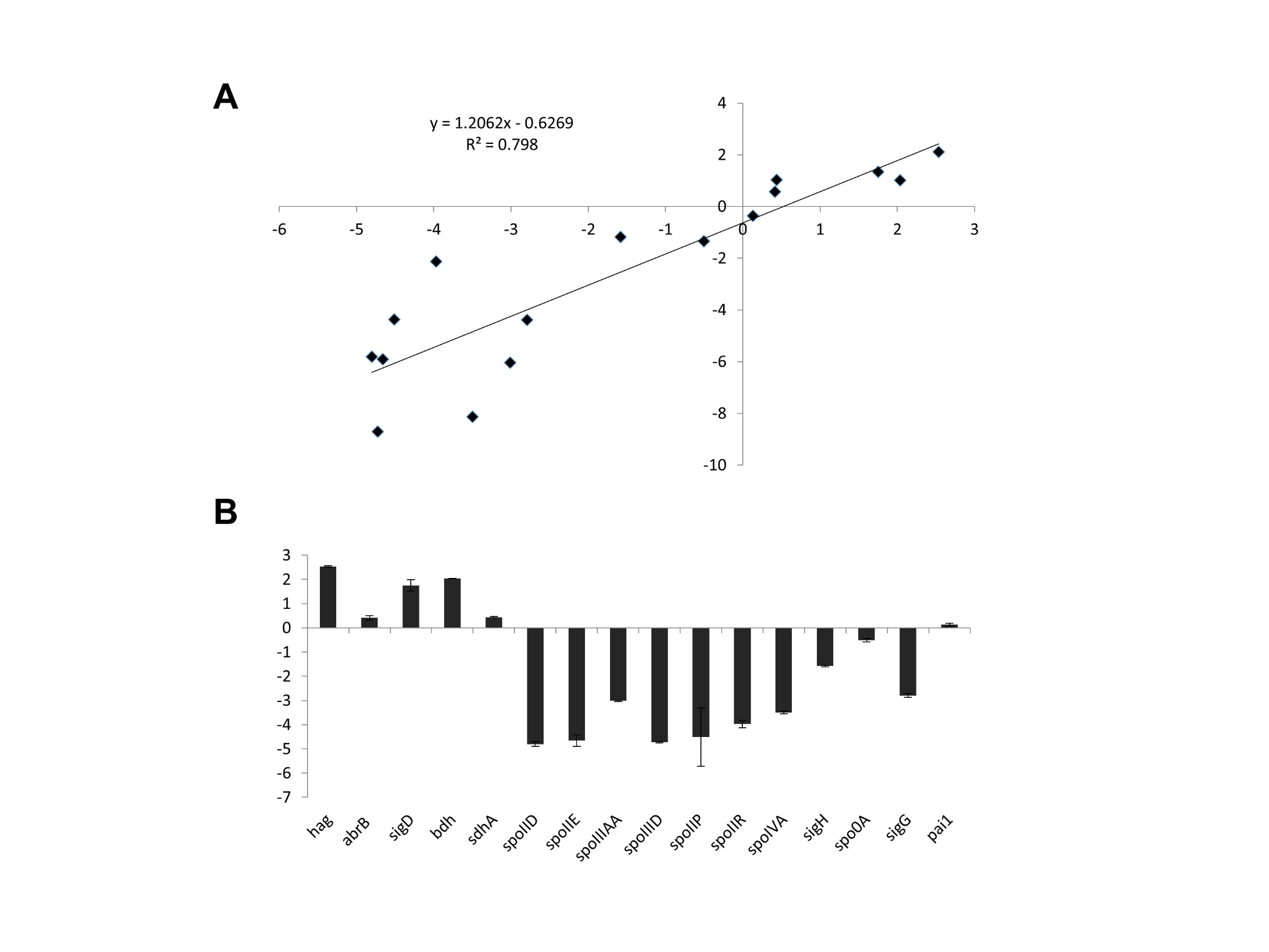


**Fig. S3. Validation of RNA-seq results using quantitative real-time PCR (qPCR).** The relative expression levels of randomly selected genes were analyzed by qPCR to validate RNA-seq results. (**A**) Log_2_ fold change measured by qPCR. (**B**) Log_2_ fold change measured by qPCR. Error bars represent standard deviation.


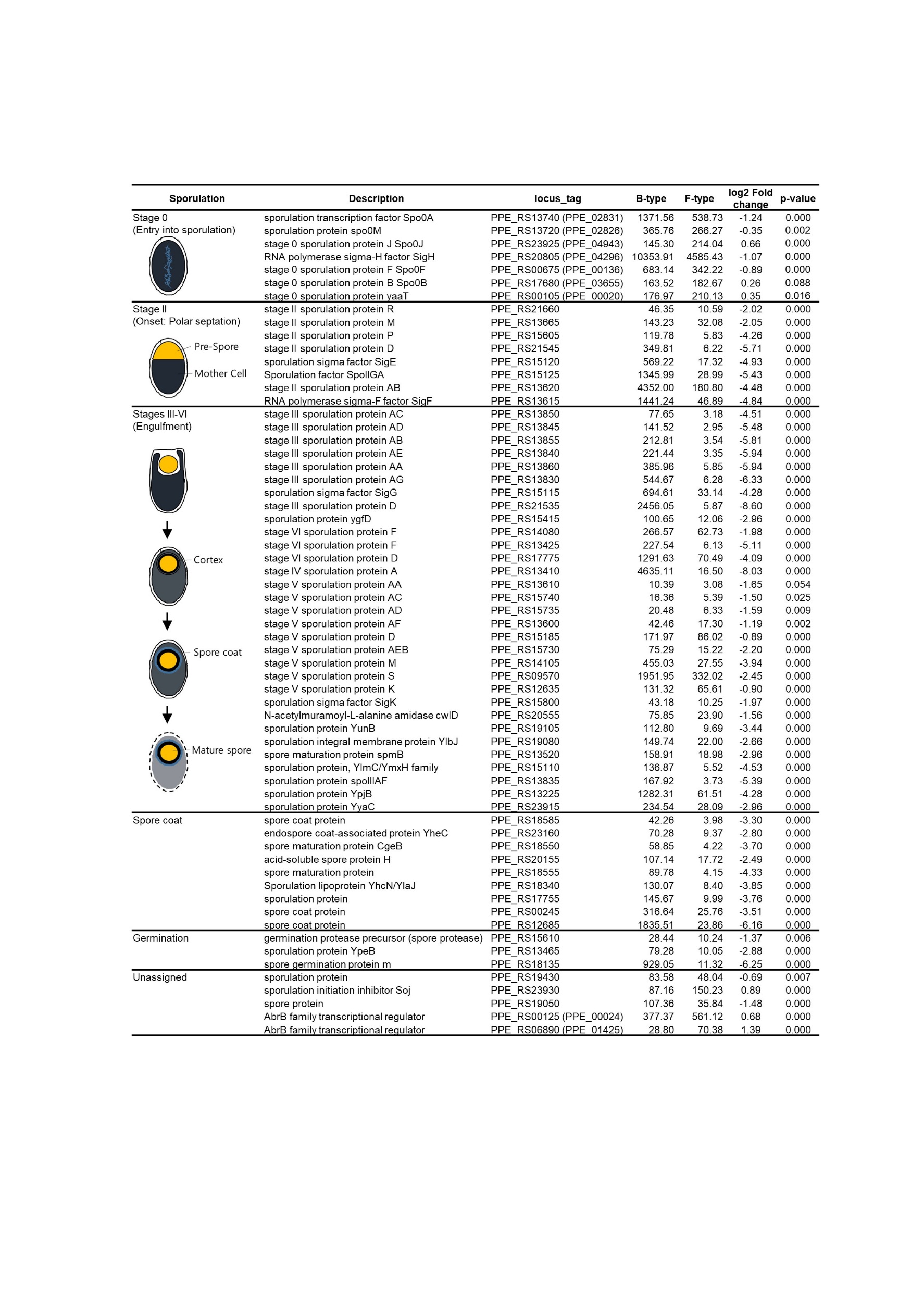


**Fig. S4.** The expression of genes known to be expressed at each stage of sporulation in F-type.
